# RAPTOR: Randomised Controlled Trial of PENTOCLO (pentoxifylline-tocopherol-clodronate) in Mandibular Osteoradionecrosis—study protocol for an open-label phase II randomised controlled superiority trial

**DOI:** 10.1186/s13063-025-08966-9

**Published:** 2025-07-24

**Authors:** Richard Shaw, Ruth Knight, Ayten Basoglu, Mandeep Bajwa, Julie Perry, Anastasios Kanatas, Stefano Fedele, Vincent Killen, Rebecca Tangney, Chris Butterworth, James McCaul, Jagtar Dhanda, Vinod Patel, Mererid Evans, Richard Jackson

**Affiliations:** 1 Liverpool Head & Neck Centre, University of Liverpool and Liverpool University Hospitals NHS Foundation Trust, Liverpool, UK; 2 Liverpool Clinical Trials Centre, University of Liverpool, Liverpool, UK; 3 Section of Oncology, School of Biosciences, Faculty of Health & Medical Sciences, University of Surrey, Guildford, UK; 4 St James Institute of Oncology, Leeds Dental Institute, Leeds General Infirmary and University of Leeds, Leeds, UK; 5 UCL Eastman Dental Institute, University College London, and NIHR University College London Hospitals Biomedical Research Centre, London, UK; 6 HaNC PPI Research Forum, Liverpool Head & Neck Centre, University of Liverpool, Liverpool, UK; 7 Liverpool University Hospitals NHS Foundation Trust, Liverpool, UK; 8 Queen Elizabeth University Hospital, Glasgow, UK; 9 Brighton and Sussex Medical School, Brighton, UK; 10 Guy’s & St Thomas’ Hospital, London, UK; 11 Cardiff University and Velindre University NHS Trust, Cardiff, Wales; 12 Department of Health Data Science, University of Liverpool, Liverpool, UK

**Keywords:** Mandible, Osteoradionecrosis, Radiotherapy, Head and neck, Cancer, PENTOCLO, Pentoxifylline, Tocopherol, Sodium clodronate, Clinical trial

## Abstract

**Background:**

Mandibular osteoradionecrosis (ORN) is a severe late radiation toxicity affecting 5–10% of patients who receive radiotherapy as part of treatment for head and neck malignancy. ORN can cause permanent disfigurement, dysfunction, pain and infection. There remains little robust evidence supporting the efficacy of medical or surgical management currently offered in clinical practice. Retrospective case series and meta-analyses of observational studies suggest that a repurposed triple drug combination pentoxifylline-tocopherol-clodronate (‘PENTOCLO’) may be effective in preventing deterioration, promoting healing and ultimately reducing the need for major reconstructive surgery.

**Methods:**

The RAPTOR trial is a phase II, open-label, multicentre, randomised controlled trial with a superiority design. Eligible subjects with mandibular ORN are randomised 1:1 to receive standard of care (Arm A) or PENTOCLO plus standard of care (Arm B) for 12 months. The primary outcome measure is time from randomisation to healing of ORN (without the need for surgery), as measured by clinical examination (confirming completely healed oral mucosa), intra-oral clinical photographs and imaging. RAPTOR has an embedded translational sample collection and a methodological component exploring the use of electronic patient-reported outcome measures (ePROM).

**Discussion:**

The RAPTOR trial is the first randomised controlled clinical trial of PENTOCLO in this clinical setting. The results of this phase II trial will provide robust preliminary evidence on its efficacy and inform the feasibility and appropriateness of a subsequent definitive trial.

**Trial registration:**

RAPTOR is registered with the ISRCTN registry effective date 11th November 2022: Clinical trial of the non-surgical management of radiotherapy damage to the lower jaw ISRCTN34217298, and also registered with the European Clinical Trials Database (Eudra-CT 2022–000728-39).

**Supplementary Information:**

The online version contains supplementary material available at 10.1186/s13063-025-08966-9.

## Administrative information

**Table:** 

Title {1}	RAPTOR: Randomised Controlled Trial of PENTOCLO (pentoxifylline-tocopherol-clodronate) in Mandibular Osteoradionecrosis – study protocol for an open-label phase II randomised controlled superiority trial
Trial registration {2a and 2b}	ISRCTN34217298 Eudra-CT 2022–000728-39
Protocol version {3}	RAPTOR Protocol V4.1 18/10/2024
Funding {4}	The RAPTOR trial is funded by the UK National Institute of Health & Care Research Efficacy and Mechanism Evaluation Programme. Award reference NIHR131050
Author details {5a}	Richard Shaw* Liverpool Head & Neck Centre, University of Liverpool and Liverpool University Hospitals NHS Foundation Trust, Liverpool, UK Ruth Knight Liverpool Clinical Trials Centre, University of Liverpool, Liverpool UK Ayten Basoglu Liverpool Clinical Trials Centre, University of Liverpool, Liverpool UK Mandeep Bajwa Section of Oncology, School of Biosciences, Faculty of Health & Medical Sciences, University of Surrey, Guildford, UK Julie Perry Liverpool Clinical Trials Centre, University of Liverpool. Liverpool, UK Anastasios Kanatas St James Institute of Oncology, Leeds Dental Institute, Leeds General Infirmary and University of Leeds, Leeds, UK Stefano Fedele UCL Eastman Dental Institute, University College London, and NIHR University College London Hospitals Biomedical Research Centre, London, UK Vincent Killen HaNC PPI Research Forum, Liverpool Head & Neck Centre, University of Liverpool, UK Rebecca Tangney Liverpool University Hospitals NHS Foundation Trust, Liverpool, UK Chris Butterworth Liverpool Head & Neck Centre, University of Liverpool and Liverpool University Hospitals NHS Foundation Trust, Liverpool, UK James McCaul Queen Elizabeth University Hospital, Glasgow, UK Jagtar Dhanda Brighton and Sussex Medical School, Brighton, UK Vinod Patel Guy's & St Thomas'Hospital, London, UK Mererid Evans Cardiff University and Velindre University NHS Trust, Cardiff, Wales Richard Jackson Department of Health Data Science, University of Liverpool, Liverpool, UK *Corresponding author
Name and contact information for the trial sponsor {5b}	**Study Sponsor:** The University of Liverpool Clinical Directorate Thompson Yates Building The Quadrangle, Brownlow Hill, Liverpool L3 5RB clinicaldirectorate@liverpool.ac.uk
Role of sponsor {5c}	The sponsor had no input to the trial design, collection, management or planned analysis, interpretation of the data, or writing of any trial reports or documents

## Introduction

### Background and rationale {6a}

Osteoradionecrosis (ORN) is defined as exposed irradiated bone that fails to heal over a period of 3 months in the absence of recurrent malignancy [1]. For many affected patients, ORN represents a devastating complication of radiotherapy, often causing intractable pain, repeated infection, jaw fracture, fistulation, disfigurement and dysfunction. This can lead to malnutrition, opiate dependency and for a proportion of patients, eventually death.

Current clinical management includes symptomatic or conservative care which includes analgesia, control of infection with chlorhexidine mouthwash, antibiotics, alleviation of any trauma caused by sharp or mobile bone sequestrum using minor outpatient interventions, using, at most, local anaesthetic. Other accepted treatment includes surgical intervention, involving complete resection of all involved bone and reconstruction as necessary [2, 3]. Surgery is a major and invasive intervention, often requiring 8–10 h and multiple surgeons, usually requiring free flap/microvascular anastomosis reconstruction and involving critical care admission and significant hospital stay; it has a high cost and significant complication rate [4]. Surgery of this nature is usually reserved as a ‘last resort’ for patients with worsening and otherwise unmanageable symptoms or increasing extent of ORN. Understandably, there is a focus on the potential for medical management of ORN that may prevent worsening and avoid such major surgery.

Medical management of mandibular osteoradionecrosis using courses of hyperbaric oxygen (HBO) or repurposed drug combinations have been explored. Recent clinical trials indicate that hyperbaric oxygen, is largely ineffective in preventing [5] or treating [6, 7] ORN. A repurposed drug combination pentoxifylline-tocopherol-clodronate (‘PENTOCLO’) [3] has been proposed as a potential pharmacological action. The pentoxifylline-tocopherol combination is thought to reverse soft tissue fibrosis induced by radiotherapy [8]. Potentiation of this combination by clodronate is indicated for osseous lesions, such as ORN of the mandible [9], without resorting to surgical resection and reconstruction. The pathophysiology and potential therapeutic opportunities in ORN have been reframed in recent years, aligned with the use of PENTOCLO, as a fibro-atrophic process. This is thought to be mediated by reactive oxygen species (ROS), a progressively fibroblastic stroma and emphasising the role of TGF-B1 [10]. Pentoxifylline, previously used to treat peripheral vascular disease, has been shown to have in-vivo anti-TNF alpha effect, to increase erythrocyte flexibility, increase vasodilation and to have anti-inflammatory effects [11]. As a single agent pentoxifylline has demonstrated some beneficial effects in soft tissue necrosis and trismus and various functional/symptomatic effects after radiotherapy in breast and head and neck cancer survivors [12]. Tocopherol acts as a ROS scavenger and an inhibitor of TGF-beta1 and procollagen gene expression, but alone has proved ineffective in the management of radiation-induced fibrosis. Combining the two agents, and exploiting their apparently synergistic mechanisms, the combination of pentoxifylline and tocopherol has demonstrated benefit in placebo-controlled trials [8] of radiation fibrosis and necrosis, although appears to need at least 12 months of continuous treatment to avoid rebound. Data on osseous lesions such as mandibular ORN has suggested adding clodronate to inhibit osteoclasts, which may potentiate the effectiveness of the protocol and this current triple regimen (termed PENTOCLO) has been used increasingly since 2005 [3]. Clodronate is a first-generation, non-nitrogenous, oral bisphosphonate, which specifically is not associated with drug-induced osteonecrosis. Clodronate reduces osteoclast activity, decreases fibroblast and macrophage proliferation, and promotes bone formation by osteoblasts. This combination has shown promise in medical management of ORN, with the necrotic bone forming a sequestrum that is eventually shed, revealing intact underlying mucosa.

There are to date no published randomised or controlled clinical trials of PENTOCLO in mandibular ORN. There is some evidence defining some therapeutic effect from retrospective case series and metanalyses. In a single-site, one-arm design, Delanian et al. [9] showed 54 patients with ORN experienced complete resolution of early ORN after a median of 9 months treatment with the PENTOCLO protocol. The available retrospective evidence has been subject to systematic review by two groups, with broadly similar conclusions. Martos-Fernandez et.al [13]. analysed 10 published series with a total of 334 patients included, finding all studies of a low or moderate methodological quality. Little numerical analysis was attempted with a largely descriptive methodology, but it was found that resolution occurred between 3 and 13 months, with 60% of patients showing clinical improvement or total healing. Healing in more advanced cases (Notani Grade 3) is less predictable and may take longer. Heterogeneity in methodology and endpoints was a barrier to further quantitative analyses. Kolokythas et al. [14] studied 7 reports of 186 patients using tighter inclusion criteria. 126 patients fully recovered or improved significantly, ORN was stable or progressive in 60 patients, with 15 patients receiving jaw resections. The estimated proportions of full resolution were 62.7%; of reduction in SOMA score were 86.5%; and of reduction of area exposed bone were 62.0%. Challenges in analysis were created by a lack of standardisation of treatment and outcome reporting. Both reviews concluded that patients tolerated PENTOCLO well, the commonest side effect being mild gastrointestinal disturbance.

### Objectives {7}

A rigorous evaluation of PENTOCLO in the management of ORN of the mandible is now necessary. The primary objective of RAPTOR is to determine if PENTOCLO triple therapy is effective in healing of mandibular ORN. This phase II trial is designed to establish the first robust signal of efficacy, an estimate of effect size, and the safety/tolerability of PENTOCLO in mandibular ORN. Additionally, a repository of blood samples from the trial will allow future studies to explore the genomic determinants of susceptibility to osteoradionecrosis [15, 16]. The results will also provide robust preliminary evidence on its efficacy and inform the feasibility and appropriateness of a subsequent definitive trial.

### Trial design {8}

The RAPTOR trial (Randomised Controlled Trial of PENTOCLO in Mandibular Osteoradionecrosis) is a phase II multicentre, open-label, randomised, controlled, superiority trial of PENTOCLO versus standard of care in patients who have mandibular ORN. The trial protocol was conceived, refined, funded and is being managed with the involvement of patient representatives. The trial has embedded translational and methodological endpoints.

## Methods: participants, interventions and outcomes

### Study setting {9}

Suitable patients will be recruited from surgical, oncology and dental follow-up clinics within UK head and neck oncology multi-disciplinary teams. The list of participating sites is available at the trial webpage [17].

#### Eligibility criteria {10}

**Fig. 1:**
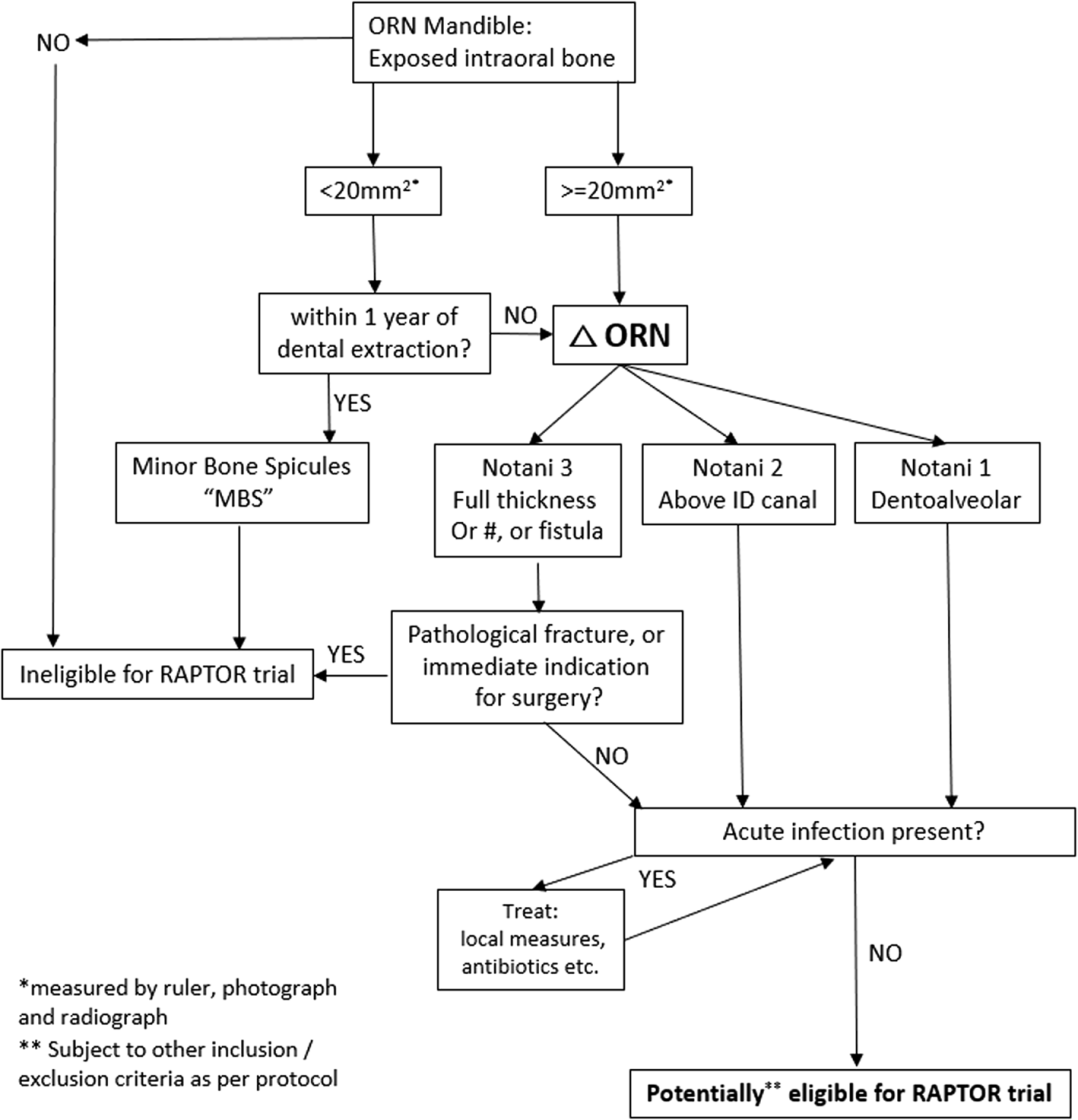
Eligibility and classification of ORN for RAPTOR trial

Patients must have had prior radiotherapy to the mandible, have diagnosed ORN of the mandible and be considered suitable for medical management. A detailed list of exclusion criteria is given in Table 1. Although definitions of ORN differ, for the purposes of the RAPTOR trial, eligible patients must have exposed mandibular bone either intra- or extra-orally. ORN is classified on clinical and radiographic grounds, from an orthopantomogram (OPT) using a modified version of the Notani Classification [1, 18], and this eligibility and classification of ORN for RAPTOR trial purposes is explained in Fig. 1. Notani class 3 cases are excluded if they have a pathological fracture on clinical assessment, including radiograph, but otherwise may be included. Patients with minor bone spicules (< 20mm^2^) within 12 months of dental extraction/surgery are very likely to heal spontaneously and thus are excluded from the RAPTOR trial. If ORN occurs spontaneously, then patients may be eligible irrespective of the surface area measurements of exposed bone.

**Table 1:** Detailed exclusion criteria RAPTOR trial

1. Cannot swallow tablets
2. Prior treatment with PENTOCLO or any element thereof within 12 months of the date of randomisation (4 months, if the PENTCLO is given for ORN prophylaxis reasons at time of dental extractions)
3. Very early ORN after surgery, defined as <20 mm^2^ exposed bone occurring within 12 months of a dental extraction or other dentoalveolar operation
4. Mandibular pathological fracture secondary to ORN
5. Mandibular pathological fracture secondary to ORN
6. Patient has had definitive resection/reconstruction for mandibular ORN—i.e. no longer has exposed necrotic bone present
7. Pregnancy
8. Lactation
9. Age <18 years
10. Acute infection at site of the necrotic bone
11. Hypersensitivity to other methylxanthines
12. Hypocalcaemia
13. Participants not willing to follow the contraceptive requirements of the protocol
14. Contraindications to PENTOCLO medications:
a. Known hypersensitivity, allergy or anaphylaxis to pentoxifylline, tocopherol or sodium clodronate
b. Treated hypotension
c. Severe coronary artery disease, defined as grade IV of the Canadian Cardiology Society Angina Grading
d. Severe cardiac arrythmia, defined as those cases with attributable syncope or heart failure associated; or those with frequent and symptomatic palpitations, breathlessness, dizziness, chest pain, weakness or fatigue.
e. Myocardial infarction within 6 months
f. Prior history of extensive retinal haemorrhage
g. Prior history of intracranial bleeding
h. Impaired renal function (creatinine clearance <30 ml/min, will be formally assessed only if U&E out of reference)
i. Severe liver failure (class B or C Pugh-Child score, will be formally assessed only if LFT values out of reference)
j. Concomitant prescription of anti-platelet agents: clopidogrel, eptifibatide, tirofiban, epoprostenol, iloprost, abciximab, anagrelide, NSAIDs, acetylsalicylates (ASA/LAS) including aspirin >75 mg*, ticlopidine, dipyridamole. (*low dose ≤75 mg aspirin is permitted)
k. Concomitant prescription of ketorolac, cimetidine, ciprofloxacin, theophylline, estramustine phosphate
l. Hereditary fructose intolerance, glucose-galactose malabsorption or sucrase-isomaltase insufficiency
m. Concomitant prescription other bisphosphonates e.g. risedronate, alendronate, aIbandronate, zoledronic acid, pamidronate, etidronate or prescription of denosusamab
15. n. Concomitant prescription of aminoglycoside antibiotics e.g. gentamicin, tobramycin, amikacin, plazomicin, streptomycin, neomycin, paromomycin

### Who will take informed consent? {26a}

Written and informed consent will be obtained from participants along with agreement of the participant to comply with the requirements of the study. Informed consent will be obtained by the site principal investigator (PI) or other appropriately qualified member of the research team who has been delegated this responsibility.

### Additional consent provisions for collection and use of participant data and biological specimens {26b}

In addition, participants will have the option to consent to their data and/or blood samples being used in future research. Consent is also required for screening procedures, explicitly detailing that DNA is to be extracted and stored from a blood test for genomic sequencing as part of ongoing radiogenomic research.

## Interventions

### Explanation for the choice of comparators {6b}

The control arm (A) reflects standard of care management of mandibular ORN in the UK. The intervention arm (B, PENTOCLO) reflects a protocol previously proposed in single-arm ORN studies [9] and that has most widely been adopted for use in ORN in the United Kingdom [3].

### Intervention description {11a}

#### Arm A (control arm, standardised supportive care (SSC))

Arm A (control arm, standardised supportive care (SSC)) may include analgesia, mouthwash, antibiotics and alleviation of local trauma, and any other appropriate measures determined by the site clinician. It specifically excludes hyperbaric oxygen and operations including surgical resection of the mandible (rim or segment). Patients with loose or sharp bone sequestrae or teeth may receive minor debridement or extractions in the outpatient setting to alleviate trauma to the oral soft tissues. This may be with the aid of local anaesthetic as needed. This excludes more major surgery to excise bone that would be carried out in an operating theatre, using saws or drills, or requiring general anaesthetic. Placebo drugs are not used.

#### Arm B (intervention arm, PENTOCLO)

Arm B (intervention arm, PENTOCLO) includes the components of SSC as described above *and*: Pentoxifylline 800 mg daily, Tocopherol 1000 mg daily, Clodronate 1600 mg days 1–5 of 7 (Monday through Friday only).

#### Patient preference phase

The trial also includes a patient preference phase which is available to all participants reaching the 12-month primary follow-up timepoint. At this point, they may elect to conclude their participation in the trial and then return to standard care outside of the trial. Alternatively, they may continue with trial treatments and 3-monthly observations. Patients who choose to continue with trial treatments may choose either to continue with their allocated arm or to switch to the alternative arm of RAPTOR. Observations will continue to the point they wish to withdraw or the point that the trial closes to recruitment. Only one such switch is permissible in-trial. The data within the patient preference phase will be analysed as a secondary endpoint and considered distinctly from the randomised portion of the trial.

### Criteria for discontinuing or modifying allocated interventions {11b}

#### Arm B: dose modifications

In the event of gastrointestinal side effects such as diarrhoea, nausea or vomiting, a divided dose regimen of sodium clodronate may first be considered. In the event of dizziness, headache, epigastric pain or nausea, a temporary 2-week dose reduction of pentoxifylline may additionally (if overlapping symptoms) or alternatively be implemented. If in either case further modification is required, halving the dose of the relevant drug may be considered in discussion with the chief investigator. Detailed specifics of administration and permitted dose modifications are detailed in full in the protocol (available at RAPTOR trial webpage [17]). Patients may discontinue treatment in Arm B in the case of unacceptable toxicity should the possible mitigations not prove tolerable. Discontinuation from PENTOCLO does not trigger discontinuation of the study altogether, and the remaining study procedures, follow-up assessment/visits, and data collection should be completed as indicated in the protocol.

#### Worsening ORN

For either arm A or B (or in patient preference phase), if ORN deteriorates to the point that medical management is no longer indicated, the patient will progress to surgical resection and reconstruction of necrotic bone. After this surgery, it is presumed that there will no longer be remaining necrotic bone to monitor on trial, so this will lead to discontinuation of the study and thus the decision will trigger the Study Completion Visit.

Additionally, patients may discontinue treatment for reasons including intercurrent illness, pregnancy, death, clinician-led assessment of patients’ condition, non-adherence, newly developed or previously unrecognised exclusion criterion.

### Strategies to improve adherence to interventions {11c}

Patient symptoms are collected using a smartphone application (ePROM) as below, which may act as a prompt to improve compliance. In arm B, drugs are dispensed at 3-monthly intervals. A telephone check on adherence is carried out at 2 weeks.

### Relevant concomitant care permitted or prohibited during the trial {11d}

#### Prescribed medicines

A complete list of prohibited concomitant medications is included in Table 1.

##### Surgery

Minor debridement or dental extractions in the outpatient setting are permitted during the trial in either arm, as described above. Formal surgical resection and or reconstruction of ORN leads to discontinuation of the study protocol at that point, also as described above.

### Provisions for post-trial care {30}

The trial also includes a patient preference phase which is available to all patients reaching the 12-month primary follow-up timepoint, as described above. After study completion, patients will return to standard of care for the management of osteoradionecrosis as clinically necessitated and determined by site clinicians.

### Outcomes {12}

The primary outcome measure is time from randomisation to healing of ORN (without the need for surgery), as measured by:

- Clinical examination by confirming completely healed oral mucosa
- Intra-oral clinical photographs
- Radiographs

Conversely, patients who demonstrate failure of treatment as below will be treated as censored observations:

- Deterioration of ORN including fracture
- A clinical indication to intervene with mandibular resection and reconstruction

Thus, either the diagnosis of complete healing at 12 months or mandibular fracture or other clinical indication to progress to surgery within 12 months both constitute a primary endpoint for that patient. Other patients whose ORN persists without the need for surgery will also reach the primary endpoint at 12 months, and any change in grade of ORN will be recorded as a secondary endpoint (as below Table 2).

**Table 2:** Secondary outcome measures RAPTOR trial

Objectives	Outcome measures	Time point(s) of evaluation
Efficacy:		
1. Deterioration of ORN	Time from randomisation to worsening of ORN as measured: • Extent of exposed bone measured in two dimensions as mm^2^ (and for some trial visits supported by clinical photograph with in-field ruler) and • Notani grade	Every 3 months following randomisation until end of trial for that patient
2. Patients’ analgesia and antibiotic use	• Drug and dose taken for pain relief in the 24-h period leading up to appointment • Days of antibiotic usage. Systemic antibiotics taken since last trial appointment (type, dose and number of days for each type)	Every 3 months following randomisation until end of trial for that patient
3. Patients’ anthropological measurements	Patient BMI (kg/m^2^) where kg is a patient weight in kilograms and m^2^ is their height in metres squared	Every 3 months following randomisation until end of trial for that patient
4. Severity of disease	Grade by “Osteonecrosis of jaw” within CTCAE v 5.0 2017	Every 3 months following randomisation until end of trial for that patient
5. Quality of life	Quality of Life (EORTC QLQ-C30 and QLQ-H&N35)	Every 3 months following randomisation until end of trial for that patient
6. Mandibular preservation	Mandibular preservation rate measured as the removal of mandibula following surgery (segmental resection, with or without reconstruction)	Every 3 months following randomisation until end of trial for that patient
Toxicity:		
7. Gastrointestinal tolerability of PENTOCLO regimen 2 weeks after commencing (Only in Arm B: PENTOCLO)	Gastrointestinal tolerability, as defined by CTCAE grading	2 weeks after commencing trial medications ± 5 working days, (may be by telephone)
8. Compliance	Evaluation of compliance to the IMP	Every 3 months following randomisation until the end of trial for that patient
9. Assessment of IMP’s combination safety	Evaluation of severe adverse events (severe AEs) and/or serious adverse events (SAEs) considered related to the study treatment	Every 3 months following randomisation until the end of trial for that patient
Exploratory:		
10. Patients’ pain and mouth function	• Pain • Eating • Mouth opening • Problems with teeth/gum • Painkillers used • Interference of these symptoms with patients’ daily activities	Every 15 days until end of trial (for that patient) Collected remotely on smartphone App

To avoid any potential risk of bias due to unblinded site investigators, OPT and clinical photographs taken with an in-field ruler (Puritan stick) are subject to a central blinded review. Training of site investigators is provided during site initiation visits and updates. In cases of trismus, clinical photographs are facilitated by intra-oral cameras which are available with disposable single use covers. Digital copies of the clinical photographs (with in-field rulers) are securely uploaded to the study database by the site staff. At the end of the trial, the central review panel will be provided with the anonymised clinical photographs by the Liverpool Clinical Trials Centre (LCTC) from baseline and 12 months (and at study completion for patients who continue beyond 12 months) for blinded assessment. The outcomes within the patient preference arm of the trial (i.e. after 12 months) will be considered and presented separately. Quality control of images and radiographs from each site is performed from the first images returned and a random sample thereafter.

The secondary outcomes measures include the determination of the impact of PENTOCLO on the deterioration of ORN, analgesia and antibiotic usage, quality of life (using EORTC QLQ-30), mandibular preservation, anthropological measurements, safety, tolerability and compliance of trial medications (complete list Table 2).

### Methodological endpoint: use of ePROM (electronic patient-reported outcome measures)

At the suggestion of patients involved in trial design, measures of pain and oral function and analgesia use are recorded every 15 days using a bespoke smartphone application. This data is entered by patients remotely upon receiving a notification, and data is subsequently populated directly to the RAPTOR trial database. A further secondary endpoint analysed will thus be the degree of completeness of data recorded using this ePROM. For patients without access to smartphones, alternative methods using computer website or telephone-based questionnaires are provided.

### Participant timeline {13}

**Fig. 2:**
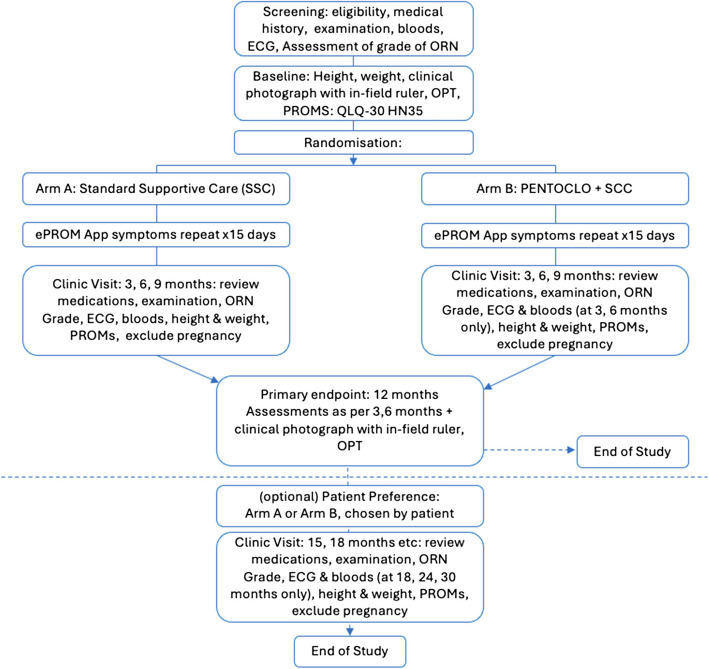
Trial schema RAPTOR trial

The schedule of enrolment. Interventions, assessments and visits are illustrated in Fig. 2 and Table 3.

**Table 3:** Participant timeline RAPTOR trial

Assessment	Screening	Baseline^a^	Randomisation	2-week check by telephone	Clinic visit schedule *(in months)*				*Optional clinic visits*^b^ *(in months)*							Study completion^b^
					**3**	**6**	**9**	**12**	**15**	**18**	**21**	**24**	**27**	**30**	**33**	
Clinic visit numbers					1	2	3	4	5	6	7	8	9	10	11	
*Accepted Variance*				± *1 week*	± *1 month*											± *1 months*
Informed consent	X															
Assessment of eligibility criteria	X															
Confirmation of eligibility	X															
Review of medical history	X	X														
Review of concomitant medications (including analgesia and antibiotic usage)	X	X			X	X	X	X	X	X	X	X	X	X	X	X
Demographics^c^	X															
General oral/head and neck examination	X	X			X	X	X	X	X	X	X	X	X	X	X	X
Intra-oral examination and assessment of ORN by Notani and CTCAE grade	X	X			X	X	X	X	X	X	X	X	X	X	X	X
ECG^d^	X				X	X		X		X		X		X		X
FBC^d^	X				X	X		X		X		X		X		X
PT and APTT^d^	X				X	X		X		X		X		X		X
UE^d^	X				X	X		X		X		X		X		X
LFT^d^	X				X	X		X		X		X		X		X
Calcium^d^	X				X	X		X		X		X		X		X
Research blood sample	X															
FSH^e^	X															
Pregnancy test^f^	X				X	X	X	X	X	X	X	X	X	X	X	X
Radiology: orthopantomogram	X							X^g^								X^h^
Patient completes training for 15-day return of ePROM^i^		X														
Height and weight measurement and BMI calculation		X			X	X	X	X	X	X	X	X	X	X	X	X
Special assay: clinical photograph with in-field ruler		X						X								X
PROM: EORTC QLQ-30 and H&N35		X			X	X	X	X	X	X	X	X	X	X	X	X
Randomisation			X													
Dispense study medication			X		X	X	X	X	X	X	X	X	X	X	X	
Collection of adverse events		X	X	X	X	X	X	X	X	X	X	X	X	X	X	X

^a^At baseline, all procedures should be done before randomisation

^b^These visits only apply to the patients who chose to continue the study after 12 months. For the rest of the patients, 12 months will be considered as study completion

^c^Gender, ethnicity

^d^During the follow-up period, these tests should be done for patients in Arm B only. During the screening, it should be done for all patients regardless of the trial arm

^e^All women other than those who have had hysterectomy and bilateral oophorectomy

^f^Women with FSH within reference range need pregnancy blood test during the screening. Patients in Arm B need to be tested throughout the treatment period

^g^May use OPT if taken for other clinical reason and within 4 months of study visit

^h^Only repeat OPT at completion if there is more than 4 months after the last OPT

^i^ePROM to be completed every 15 days (or facilitated using website/research staff every 30 days)

### Sample size {14}

Literature-based estimates of the use of PENTOCLO give a 12-month healing rate of approximately 60% and it is considered that this would have to demonstrate an improvement over a 40% rate in the control arm (equivalent to a hazard ratio of 0.56). Using a one-sided alpha level of 0.1 and a power of 90%, then a total of 78 events are required. Including a 5% rate for patient attrition and based on estimated recruitment rates, it is anticipated that 120 patients are required to obtain the events required.

### Recruitment {15}

Approximately 15 UK head and oncology centres will recruit to the trial. Additionally, patient-facing material will be disseminated using social media and by circulating to head and neck oncology patient support networks, giving access to their nearest open centre.

## Assignment of interventions: allocation

### Sequence generation {16a}

Participants will be randomised to receive either arm A, standard supportive care (SSC), or arm B, PENTOCLO + SSC, (in a ratio of 1:1).

### Concealment mechanism {16b}

Participants and site staff will be unblinded as to allocation.

### Implementation {16c}

Participants will be randomised via a secure (24-h) web-based randomisation system controlled centrally by the trials centre. Randomisation lists will be generated in advance by a member of the trials unit team and will be stratified by study site using permuted blocks. This system is generated centrally by the trials unit using a computer algorithm and is concealed from the investigators and research teams/trial management group.

## Assignment of interventions: blinding

### Who will be blinded {17a}

Assessment of the primary endpoint is blinded, as de-identified clinical photographs and radiographs will be assessed by a remote, blinded, expert panel of assessors.

### Procedure for unblinding if needed {17b}

Not applicable, as participants and site staff will be unblinded.

## Data collection and management

### Plans for assessment and collection of outcomes {18a}

All data will be managed as per local Liverpool Clinical Trials Centre (LCTC) processes and in line with all relevant regulatory, ethical and legal obligations. The bespoke Case Report Forms (CRF) will be considered the source document for data where no prior record exists. A description of study instruments and classifications used are in the protocol available at the RAPTOR trial webpage. The CRFs are not available in a publishable form or document (and therefore cannot be appended or referenced) but details of their content are available by direct contact with the chief investigator.

### Plans to promote participant retention and complete follow-up {18b}

Retention of patients is encouraged by using 3-monthly trial visits, through telephone support by participating centres, and through prompting by use of the RAPTOR ePROM bespoke smartphone application. Discontinuation from trial intervention does not mean discontinuation of the study altogether, and the remaining study procedures, follow-up assessment/visits and data collection should be completed as indicated in the protocol (unless consent is specifically withdrawn). The exception for this is for surgical resection of ORN, and when this is performed, it will trigger a study completion visit irrespective of timepoint, as described above.

### Data management {19}

Data management procedures are described in detail in the protocol, available at the RAPTOR trial webpage [17].

### Confidentiality {27}

This trial will collect personal data (e.g. participant names), including special category personal data (i.e. participant medical information) and this will be handled in accordance with all applicable data protection legislation. This data will be handled confidentially and securely, and full details are within the protocol available at the RAPTOR trial webpage [17].

### Plans for collection, laboratory evaluation and storage of biological specimens for genetic or molecular analysis in this trial/future use {33}

In addition to the participant’s other blood samples, one blood sample is collected at the screening appointment, and stored by LCTC. The purpose of the sample is to create a bioresource for subsequent radiogenomic studies. The translational aspects of the study are exploratory and are not included in the trial endpoints. The translational work will be subject to the translational study report and not be part of the RAPTOR trial analysis plan.

## Statistical methods

### Statistical methods for primary and secondary outcomes {20a}

The primary outcome is time to healing. Healing rates will be estimated using the method of Kaplan and Meier. Comparisons across treatment groups will be performed using a log-rank test. Further analysis adjusting for key demographic and clinical covariates will be performed using a Cox proportional hazards model. The primary analysis will be carried out on the full analysis set, which will be defined on the intention to treat principle, retaining patients in their initially randomised groups irrespective of any protocol violations. Analysis of the primary outcome will be assessed using 1-tailed 0.1 level, as is consistent with the type I alpha level used in the study design. The one-sided test is employed in order to limit sample size and in the light of previous systematic reviews where no detriment to healing from PENTOCLO has been reported. All analyses of secondary outcomes will use the nominal *p* < 0.05 level to determine statistical significance. Full details of all planned analyses will be specified in a statistical analysis plan, which is provided as Appendix 1 (supplementary data).

### Interim analyses {21b}

Interim analyses are not planned.

### Methods for additional analyses (e.g. subgroup analyses) {20b}

Additional or subgroup analyses are not planned, save for analysis of the patient preference phase, which will be largely descriptive and will be considered separately from the primary endpoint, as it is non-randomised data.

### Methods in analysis to handle protocol non-adherence and any statistical methods to handle missing data {20c}

Missing data are expected to be minor, and final analyses are planned to be carried out on a complete case basis. As much information as possible will be collected about the reasons for missing outcome data; this will be used to inform any imputation approaches employed in the analysis. If substantial missing data (> 10%) are observed and it is considered appropriate upon review, then multiple imputation using chained equations will be applied.

### Plans to give access to the full protocol, participant-level data and statistical code {31c}

The full protocol and analysis plan is available at the RAPTOR trial webpage [17]. All participant-level data will be published in the main trial report.

## Oversight and monitoring

### Composition of the coordinating centre and trial steering committee {5d}

#### Trial Management Group (TMG)

The TMG comprises the chief investigator, other lead investigators (clinical and non-clinical), patient representative, and members of the LCTC. The TMG will be responsible for the day-to-day running and management of the trial and will meet at least three times per year.

#### Trial Steering Committee (TSC)

The Trial Steering Committee will consist of an independent chairperson, other independent experts in the field of oral cancer, a statistician and at least one patient.

representative. The role of the TSC is to provide overall supervision for the trial and provide advice through its independent Chairman. The ultimate decision for the continuation of the trial lies with the TSC.

### Composition of the data monitoring committee, its role and reporting structure {21a}

#### Independent Data and Safety Monitoring Committee (IDSMC)

The Independent Data and Safety Monitoring Committee (IDSMC) consists of an independent chairperson in a related area of expertise, plus 2 independent members, one of whom is also an expert in a related area and another who is an expert in medical statistics. The IDSMC, which is constituted according to a published charter within guidelines established by the funder (NIHR) will be responsible for reviewing and assessing recruitment, interim monitoring of safety and effectiveness, trial conduct and external data. The IDSMC was convened before the trial opened to recruitment and will then define the frequency of subsequent meetings (at least annually). The IDSMC will provide a recommendation to the TSC concerning the continuation of the study.

### Adverse event reporting and harms {22}

Adverse events will be reported up to the point of primary endpoint and continued for patients remaining within patient preference phase. All nonserious adverse events (AE)/adverse reactions (AR), whether expected or not, will be recorded in the relevant page of the case report form. Serious adverse reactions (SARs), serious adverse events (SAEs) and suspected unexpected serious adverse reactions (SUSARs) should be reported within 24 h of the local site becoming aware of the event. Further details over the trial procedures pertaining to SAE, SAR and SUSARs are provided in detail in the RAPTOR protocol, available at the RAPTOR trial webpage [17].

### Frequency and plans for auditing trial conduct {23}

Monitoring is conducted to ensure protection of patients participating in the trial and all aspects of the trial (procedures, laboratory, trial intervention administration and data collection) are of high quality and conducted in accordance with sponsor and regulatory requirements. A detailed Trial Monitoring Plan is detailed within the full protocol available at RAPTOR trial webpage [17].

### Plans for communicating important protocol amendments to relevant parties (e.g. trial participants, ethical committees) {25}

Amendments to the trial protocol will be cascaded to all sites and trial investigators after appropriate ethical and regulatory approvals. Where relevant, amendments will additionally be communicated to trial participants. A chronology of protocol amendments is detailed in the relevant section of the protocol.

### Dissemination plans {31a}

The results from different participating sites will be analysed together and published as soon as possible. The findings of the trial will be summarised on the study web pages. Additionally, summaries of the main findings, in plain English form, will be provided to trial participants. The results generated from this Phase II trial may be used to guide the design and requirement for a subsequent phase III trial.

## Trial status

The RAPTOR trial is open and recruiting. The current Protocol Version Number is Protocol V4.1 18/10/2024). Recruitment began on 27/4/2023 and the study is expected to complete in 2027.

## Supplementary Information

Supplementary Material 1: Appendix 1. Statistical Analysis Plan RAPTOR Trial

Supplementary Material 2: Appendix 2. Patient Information and Consent RAPTOR Trial

Supplementary Material 3: Appendix 3. RAPTOR GP Letter.

## Data Availability

Data will be published as soon as a possible and made available publicly. There are no contractual limitations on the availability of data to investigators.

## Abbreviations

RAPTOR: Randomised Controlled Trial of PENTOCLO in Mandibular Osteoradionecrosis
PENTOCLO: Drug combination: pentoxifylline, tocopherol and sodium clodronate
ORN: Osteoradionecrosis
ePROM: Electronic patient-reported outcome measure
OPT: Orthopantomogram (jaw x-ray)
LCTC: Liverpool Clinical Trials Centre
REC: Research ethics committee
IRAS: Integrated Research Application System
NIHR: National Institute for Health and Care Research
