# Supplementary material for: RAPTOR: Randomised Controlled Trial of PENTOCLO (pentoxifylline-tocopherol-clodronate) in Mandibular Osteoradionecrosis—study protocol for an open-label phase II randomised controlled superiority trial
Source: Trials. 2025 Jul 24;26:254. doi: 10.1186/s13063-025-08966-9 (PMC12291413; doi:10.1186/s13063-025-08966-9)
Supplement: Supplementary file 1 — Supplementary Material 1: Appendix 1. Statistical Analysis Plan RAPTOR Trial [file 13063_2025_8966_MOESM1_ESM.pdf]

RAPTOR: Randomised Controlled Trial of PENTOCLO* in Mandibular Osteoradionecrosis

***(Pentoxifylline, Tocopherol & Clodronate)**

EudraCT Ref: 2022-000728-39

ISRCTN: 34217298

Statistical Analysis Plan

Version 1.0 06/11/2023

DRAFT

	ORIGINATED BY	QC PERFORMED BY	APPROVED BY
**Name**	Liam Whittle	<QC>	Ruth Knight
**Title**	Trial Statistician	<Job title>	Lead Statistician
**Date**	06/11/2023		
**Protocol Version and Date**	RAPTOR Protocol V3.0 07/08/2023		

1 Approval and Agreement

SAP Version Number being approved: v1.0

Trial Statistician

Name Liam Whittle

Signed* Liam Whittle (box SIGN 4YR6PL55-15X55YXK) Date Nov 7, 2023

***Signature should be evidenced via physical signature (wet-ink or electronic).**

Senior Statistician

Name Ruth Knight

Signed* Ruth Knight (box SIGN 4Y366J34-15X55YXK) Date 6 Nov 2023

***Signature should be evidenced via physical signature (wet-ink or electronic).**

Chief Investigator/clinical lead

Name Richard Shaw

Signed* Richard Shaw (box SIGN 46ZJP9J8-15X55YXK) Date Nov 7, 2023

***Signature should be evidenced via physical signature (wet-ink or electronic).**

DRAFT

2 Table of Contents

1 Approval and Agreement....2
2 Table of Contents....3
3 Glossary....5
4 Roles and Responsibilities....6
5 Statement of Compliance....6
6 Introduction....7
7 Background and Rationale....7
8 Objectives....7
9 Study Design....8
9.1 Overall study design....8
9.2 Blinding....8
10 Consent process....8
11 Study population....9
11.1 Inclusion criteria....9
11.2 Exclusion criteria....9
11.3 Removal of participants from intervention or follow-up....10
12 Method of assignment to intervention....11
13 Schedule of assessments....12
14 Interventions....12
15 Listing of Outcomes....12
15.1 Primary outcome(s)....12
15.2 Secondary outcomes....13
15.2.1 Exploratory outcome....14
16 Sample size calculation....14
17 Study Framework....14
18 Timing and Objectives of Analyses....14
18.1 Interim Reporting....14
18.1.1 Reports to Independent Oversight Committees....14
18.1.2 Assessments of progression criteria....15
18.1.3 Formal Interim Analyses....15
18.2 Final Analysis....15
19 Disposition of Participants....15
19.1 Screening, eligibility and recruitment....16

DRAFT

19.2 Post randomisation discontinuations 19
20 Protocol Deviations 19
21 Unblinding 20
22 Analysis Datasets 20
22.1 Efficacy Analyses 20
22.2 Safety Analyses 20
23 Baseline Characteristics 20
24 Compliance with Interventions 22
25 Analysis of Outcomes 22
25.1 Interim Reporting 22
25.1.1 For reports to independent oversight committees 22
25.1.2 Assessment of progression criteria 22
25.1.3 Formal Interim Analysis 22
25.2 Final Analysis 22
25.2.1 Levels of significance and multiplicity 22
25.2.2 Primary Outcome(s) 22
25.2.3 Secondary Outcomes 24
25.2.4 Exploratory outcomes 33
26 Safety Evaluations 35
27 Additional Analyses 35
28 Document History 36
29 References 37
29.1 Non-standard statistical methods 37
29.2 Data Management Plan 37
29.3 Trial Master File and Trial Statistical File 37
29.4 Other Standard Operating Procedures to be adhered to 37
29.5 Other references 37

DRAFT

3 Glossary

ASA/LAS	Acetylsalicylates
ADL	Activities of daily living
AE	Adverse event
AR	Adverse reaction
BMI	Body mass index
CRF	Case report form
CTCAE	Common Terminology Criteria for Adverse Events
EORTC	European Organisation for Research and Treatment of Cancer
EudraCT	European Clinical Trials Database
HR	Hazard Ratio
IDSMC	Independent Data and Safety Monitoring Committee
IMP	Investigational medicinal product
IOC	Independent Oversight Committee
IQR	Inter-quartile range
ITT	Intention to treat
LCTC	Liverpool Clinical Trials Centre
LFT	Liver function test
NSAID	Non-steroidal anti-inflammatory drugs
OR	Odds ratio
ORN	Osteoradionecrosis
PENTOCLO	Pentoxifylline, Tocopherol & Clodronate
QLQ	Quality of Life Questionnaire
QoL	Quality of Life
RAPTOR	Randomised Controlled Trial of PENTOCLO in Mandibular Osteoradionecrosis
SAE	Serious adverse event
SAP	Statistical Analysis Plan
SAR	Statistical Analysis Report
SD	Standard deviation
SSC	Standard Supportive Care
TSC	Trial Steering Committee
U&E	Urea and electrolytes

DRAFT

4 Roles and Responsibilities

Liam Whittle: Trial Statistician (Liverpool Clinical Trials Centre, University of Liverpool) drafted the Statistical Analysis Plan (SAP) based on the trial protocol and Dr Ruth Knight: Statistical Lead (Liverpool Clinical Trials Centre, University of Liverpool) reviewed, amended and approved the SAP. Professor Richard Shaw: Professor of Head & Neck Surgery, Honorary Consultant in Oral Maxillofacial Surgery (University of Liverpool Cancer Research Centre, Aintree University Hospitals NHS Foundation Trust) Chief Investigator also approved the SAP.

5 Statement of Compliance

This SAP provides a detailed and comprehensive description of the pre-planned analyses for the study "Randomised Controlled Trial of PENTOCLO* in Mandibular Osteoradionecrosis *(Pentoxifylline, Tocopherol & Clodronate)". The planned statistical analyses described within this document are compliant with those specified in brief within the RAPTOR protocol V3.0 07/08/2023.

The purpose of the plan is to:

a. Ensure that all analyses are appropriate for the aims of the trial, reflect good statistical practice, and minimise bias by preventing inappropriate post hoc analyses.
b. Ensure that the analyses performed are consistent with the conditions of the protocol.
c. Explain in detail how the data will be handled, covariates derived and analysed to enable the analysis to be replicated, if necessary.

This study is carried out in accordance with the World Medical Association Declaration of Helsinki (1964) and the Tokyo (1975), Venice (1983), Hong Kong (1989) and South Africa (1996) amendments and will be conducted in compliance with the protocol, Clinical Trials Research Centre (CTRC) Clinical Trials Unit (CTU) Standard Operating Procedures (SOPs) and EU Directive 2001/20/EC, transposed into UK law as the UK Statutory Instrument 2004 No 1031: Medicines for Human Use (Clinical Trials) Regulations 2004.

DRAFT

6 Introduction

A confidential report will be provided for the independent oversight committee (IOC).

The results of the final analysis described within this statistical analysis plan will be contained within a statistical analysis report. This report will be used as the basis of the primary research publications according to the study publication plan. Where analyses are presented which are not included in the SAP, justification as to their inclusion must be provided.

This study is a clinical trial of medicinal products and is registered on the European Clinical Trials Database (EudraCT) database. The statistical analysis plan has been developed to support the posting of results on the EudraCT system. This is a regulatory requirement which should be fulfilled within 12 months after the end of the study as defined within the clinical trial protocol.

All analyses are performed with standard statistical software (SAS version 9.4 or later). The finalised analysis datasets, programs and outputs will be archived following Good Clinical Practice guidelines and SOP GE012 Archiving Procedures in LCTC.

7 Background and Rationale

Osteoradionecrosis (ORN) is defined as exposed irradiated bone that fails to heal over a period of 3 months in the absence of recurrent malignancy[(1)]. It is the most feared complication of radiotherapy often causing repeated infection, jaw fracture, fistula, malnutrition, opiate dependency and sometimes death. Current practice includes symptomatic/conservative care which is reported to resolve between 25-44% of cases[(2)].

Recent clinical trials indicate that the main historical alternative to surgery, hyperbaric oxygen, is ineffective in preventing[(3)] or treating[(4)] ORN. Some clinicians have started using PENTOCLO 'ad hoc', and with some variations in protocol, prior to planned surgery[(5)], further compounding costs to the NHS. There is an urgent need for rigorous trials of PENTOCLO therapy in the management of ORN of the mandible. This phase II proposal will establish the first robust signal of efficacy, an estimate of effect size, and the safety/tolerability of PENTOCLO in mandibular ORN. Additionally, it will inform the need for and help with design of any subsequent phase III trial.

8 Objectives

RAPTOR aims to establish the value of the repurposed drug combination Pentoxifylline, Tocopherol & Clodronate (termed PENTOCLO) in treating ORN of the mandible. The primary objective is to assess

whether there is sufficient evidence that PENTOCLO is superior to standard supportive care in healing of mandibular ORN. Further objectives include evaluation of the impact of PENTOCLO on pain and mouth function, ability to receive treatment and control the disease, analgesia and antibiotic use, anthropological measurements, severity of disease, overall quality of life, mandibular preservation, and associated toxicity. Please refer to the "Objectives" section of the protocol for further details on the study objectives.

9 Study Design

9.1 Overall study design

This is a phase II, unblinded, randomised, multi-centre trial of the of the repurposed drug combination PENTOCLO, with blinded review of primary endpoint, with a 1:1 allocation ratio of PENTOCLO vs standard supportive care in patients with ORN of the mandible who have been cured of head and neck cancer and have received radiotherapy.

9.2 Blinding

RAPTOR is an unblinded (open-label) trial: neither patients, site investigators or trial site staff are blinded as to allocation. The integrity and robustness of the primary endpoint measures are however, maintained by assessment of healing and area of exposed bone using clinical photographs and orthopantomograms. These images form the basis of assessment by a remote expert panel which is blinded to allocation of trial arm.

10 Consent process

Written informed consent will be sought from patients who will be approached by the study team and invited to consider participation.

Patients will be approached by a member of the local research team during their initial referral appointment or during routine follow-up. A written information sheet that forms part of the ethically approved Information Sheet and Consent form will be provided. This includes a detailed explanation of the study and makes clear that the rights and welfare of the participants will be protected; it will be emphasised that consent may be declined or withdrawn at any time in the future without the quality of care being adversely affected. The research staff will facilitate verbal discussions about the research and the consent process, as well as providing answers to any questions that arise.

DRAFT

After verbal and written information has been provided, the individual seeking consent will ensure that the patient has fully understood all the information and will ask if they are happy to consent to participation in the trial. Please refer to the "Informed Consent" section of the protocol for further details on the consent process.

11 Study population

11.1 Inclusion criteria

Patients eligible for the trial must comply with all of the following at randomisation:

1. A diagnosis of mandibular ORN (as specified in Appendix A of the protocol)
2. Patients considered suitable for medical management
3. Written and informed consent obtained from participant and agreement of participant to comply with the requirements of the study

11.2 Exclusion criteria

Any patient meeting any of the criteria listed below at baseline will be excluded from study participation:

1. Cannot swallow tablets
2. Prior treatment with PENTOCLO or any element thereof within 1 year of the date of randomisation
3. Very early ORN (<20 mm^2 exposed bone) occurring within 12 months of a dental extraction or other dentoalveolar operation ('Minor Bone Spicules' see flowchart below)
4. Mandibular pathological fracture secondary to ORN
5. Indication for mandible resection- i.e. patient for whom the severity of their ORN symptoms already constitute an indication for mandible resection and reconstruction. Typically, these symptoms will include severe pain, repeated infections, significant mobile pathological fracture or distressing fistula)
6. Patient has had definitive resection / reconstruction for mandibular ORN – i.e. no longer has exposed necrotic bone present
7. Pregnancy
8. Lactation
9. Age <18 years
10. Acute infection at site of the necrotic bone. The acute phase of infection should be controlled by appropriate antibiotics and other measures prior to enrolling the patient into the trial
11. Hypersensitivity to other methylxanthines
12. Hypocalcaemia
13. Participants not willing to follow the contraceptive requirements of the protocol
14. Contraindications to PENTOCLO medications:
    a. Known hypersensitivity, allergy or anaphylaxis to pentoxifylline, tocopherol or sodium clodronate

b. Treated hypotension
c. Severe coronary artery disease defined as grade IV of the Canadian Cardiology Society Angina Grading (see Appendix B of the protocol)
d. Severe cardiac arrhythmia, defined as those cases with attributable syncope or heart failure associated; or those with frequent and symptomatic palpitations, breathlessness, dizziness, chest pain, weakness or fatigue
e. Myocardial infarction within 6 months
f. Prior history of extensive retinal haemorrhage
g. Prior history of intracranial bleeding
h. Impaired renal function (Creatinine clearance <30 ml/minute, will be formally assessed only if urea and electrolytes (U&E) out of reference)
i. Severe liver failure (class B or C Pugh-Child Score, will be formally assessed only if liver function test (LFT) values out of reference)
j. Concomitant prescription of anti-platelet agents: clopidogrel, eptifibatide, tirofiban, epoprostenol, iloprost, abciximab, anagrelide, non-steroidal anti-inflammatory drugs (NSAIDs), acetylsalicylates (ASA/LAS) including aspirin >75 mg*, ticlopidine, dipyridamole. (*low dose =<75 mg aspirin is permitted)
k. Concomitant prescription of ketorolac, cimetidine, ciprofloxacin, theophylline, estramustine phosphate
l. Hereditary fructose intolerance, glucose-galactose malabsorption or sucrase-isomaltase insufficiency
m. Concomitant prescription other bisphosphonates e.g. risedronate, alendronate, albandronate, zoledronic acid, pamidronate, etidronate or prescription of denosusamab
n. Concomitant prescription of aminoglycoside antibiotics e.g. gentamicin, tobramycin, amikacin, plazomicin, streptomycin, neomycin, paromomycin

11.3 Removal of participants from intervention or follow-up

Participants may discontinue treatment for reasons including, but not limited to:

- Participant-led i.e. request by the participant
- Unacceptable toxicity, although dose reductions are a possible mitigation that should be explored (see Section 11 of the protocol)
- Intercurrent illness preventing further treatment
- Pregnancy
- Death
- Clinician-led:
    - Any change in the participant's condition that justifies the discontinuation of treatment in the clinician's opinion
    - Reasons of non-adherence or non-compliance with treatment or other trial procedures
    - Participant meets an exclusion criterion (either newly developed or not previously recognised)

DRAFT

Discontinuation from trial intervention does not mean discontinuation of the study altogether, and the remaining study procedures, follow-up assessment/visits and data collection should be completed as indicated in the protocol (unless consent is specifically withdrawn). The exception for this is for surgical resection of ORN, and when this is performed, it will trigger a Study Completion Visit irrespective of timepoint.

Participants are free to withdraw from follow-up at any time without providing a reason, though a reason should be recorded if one is given. Those who wish to withdraw from further follow-up will have the data collected up to the point of that withdrawal included in the analyses. The participant will not contribute further data to the study and LCTC should be informed via email to LCTC and via completion of a Withdrawal Case Report Form (CRF) to be returned to LCTC within 7 days.

In the case of ongoing adverse events, participants should be given appropriate care under medical supervision until the symptoms of any adverse event resolve or the participant's condition becomes stable. Any SAEs will be notifiable to LCTC via processes detailed in Section 11 of the protocol even if a participant has withdrawn from follow-up.

12 Method of assignment to intervention

Patients are randomised in a 1:1 ratio using a secure web-based randomisation programme controlled centrally by LCTC to ensure allocation concealment. This web-based tool will be accessed by research team at site. This system is generated centrally by the LCTC using a computer algorithm concealed from the investigators and research teams/trial management group.

Randomisation lists shall be produced by a statistician at the LCTC prior to the recruitment of the first patient. Patients shall be randomised using a 1:1 ratio. Lists shall be produced based on the principle of randomly permuted blocks with random block varying sizes. Lists will be stratified by study site.

Patient allocations will be irrevocably generated upon completion of the web-based randomisation form by a delegated member of the trial research team. Allocation concealment will be ensured as the service will not release the randomisation code until the patient has been recruited into the trial; this takes place after all baseline measurements have been completed.

13 Schedule of assessments

The schedule of assessments for the trial can be found in study protocol, in section 10.8. Each participant will be followed up for a minimum of 1 year and a maximum of 3 years, and the duration of the trial for recruitment will be 2 years.

14 Interventions

The exploratory intervention for the trial is the repurposed drug combination PENTOCLO. Participants will be randomised between arm A, the control arm and arm B, the experimental arm. Participants in arm A will receive standard supportive care as described in section 8.2 of the protocol, and participants in arm B will receive standard supportive care and PENTOCLO as described in section 8.3 of the protocol.

15 Listing of Outcomes

15.1 Primary outcome(s)

The primary outcome measure is time to healing, measured as the time from randomisation to confirmed healing (without the need for surgery) as measured by:

- clinical examination by confirming completely healed oral mucosa
- intra-oral clinical photographs

Conversely, participants who demonstrate failure of treatment, either:

- deterioration of ORN (see protocol Appendix A), including fracture
- a clinical indication to intervene with mandibular resection & reconstruction

will be treated as censored observations.

To avoid any potential risk of bias due to unblinded site investigators, orthopantomograms and clinical photographs will be taken with an in-field ruler (Puritan stick) and subject to a central blinded review. In cases of trismus, clinical photographs are facilitated by intra-oral cameras which are available with disposable single use covers. Digital copies of the clinical photographs will be securely uploaded to the study database by the site staff. At the end of the trial, the central review panel will be provided with the anonymised clinical photographs by the LCTC from baseline and 12 months (and at study completion for patients who continue beyond 12 months) for blinded assessment.

Time point: Every 3 months (+/- 1 month) following randomisation until end of trial for that participant.

15.2 Secondary outcomes

The secondary outcomes in this study are:

Time from randomisation to worsening of ORN: two measures of worsening will be considered every 3 months post-randomisation until the end of the trial for that patient: (i) extent of exposed bone in two dimensions as mm^2 (and for some trial visits supported by clinical photograph with in-field ruler); and, (ii) Notani grade. A participant's ORN will be considered to have worsened if it has worsened on either of these measures.

Analgesia use: every 3 months post-randomisation until the end of trial for that patient details of the analgesia used in the preceding 24-hour period will be recorded. This will include the drug and dosage.

Antibiotic use: every 3 months post-randomisation until end of trial for that patient details of any antibiotic usage specifically related to ORN since the previous trial visit will be recorded. This will include the type of systemic antibiotic, the dose and the number of days for each type.

Anthropological measurements: every 3 months post-randomisation until the end of trial for that patient participants' height (m) and weight (kg) measurements will be recorded and from these measurements, body mass index (BMI) (kg/m^2) will be derived.

Disease severity using Common Terminology Criteria for Adverse Events (CTCAE) Grade by "Osteonecrosis of jaw": measured by intra-oral examination every 3 months post-randomisation until end of trial for that patient.

European Organisation for Research and Treatment of Cancer (EORTC) Quality of Life Questionnaire (QLQ)-C30 and QLQ-H&N35 scores: recorded every 3 months post-randomisation until the end of trial for that patient using the EORTC QLQ-C30 questionnaire with the QLQ-H&N35 module.

Mandibular preservation rate: number of participants who have not undergone surgery to remove the mandible by the time of their 12-month appointment.

Gastrointestinal tolerability of PENTOCLO regimen as defined by CTCAE grading: number of and grade of gastrointestinal adverse events (as defined by CTCAE grading) experience during the first two weeks post commencement of trial medications for participants in Arm B: SSC and PENTOCLO only.

Investigational Medicinal Product (IMP) compliance: every 3 months post-randomisation until the end of the trial for that patient participants' compliance to the IMP will be determined by investigating recorded instances of premature discontinuation.

Severe adverse events (severe AEs) and serious adverse events (SAEs) related to the study treatment: number of and grade of severe adverse events (severe AEs) and/or serious adverse events (SAEs) considered related to the study treatment and experienced during the trial records every 3 months post-randomisation until the end of trial for that patient for participants in Arm B: SSC and PENTOCLO only.

DRAFT

15.2.1 Exploratory outcome

An exploratory outcome of this study is:

eProm self-reported participant measures: recorded every 15 days post-randomisation and comprising participants' self-reported pain medication use in the previous 24 hours, self-reported participant measures relating to pain, eating, mouth opening, problems with teeth/gm, amount of painkiller taken and interference of symptoms with participants' daily activities.

16 Sample size calculation

The primary endpoint is the time-to-healing measured as a time-to-event outcome. The definition is time from randomisation to healing of ORN.

Current estimates of the use of PENTOCLO give a 12-month healing rate of approximately 60% and it is considered that this would have to demonstrate an improvement over a 40% rate in the control arm (equivalent to a hazard ratio (HR) of 1.79). Using a one-sided alpha level of 0.1 and a Power of 90% then a total of 78 events are required. Including a 5% rate for patient attrition and based on estimated recruitment rates, it is estimated that 120 patients are required to obtain the events required.

Recruitment is planned to take place from 15 contributing sites which recruit at an average rate of 5.8 patients/year and be opened to recruitment at a rate of 1 site/month. A total of 24 months recruitment is required to recruit the 120 patients required.

17 Study Framework

The overall objective is to determine if the use of PENTOCLO is superior to standard care with respect to 12-month healing rate of ORN.

18 Timing and Objectives of Analyses

18.1 Interim Reporting

18.1.1 Reports to Independent Oversight Committees

There are no formal stopping rules based on efficacy or futility of the primary endpoint. Summaries of the accumulating data will be prepared at regular intervals (at least annually) for review by an Independent Data and Safety Monitoring Committee (IDSMC). These summaries will be prepared at the LCTC. The IDSMC will be asked to give advice on whether the accumulated data from the trial, together with results from other relevant trials, justifies continuing recruitment of further patients or

DRAFT

further follow-up. A decision to discontinue recruitment, in all patients or in selected subgroups will be made only if the result is likely to convince a broad range of clinicians including participants in the trial and the general clinical community.

18.1.2 Assessments of progression criteria

N/A

18.1.3 Formal Interim Analyses

Formal interim analyses of the accumulating data will not be performed.

18.2 Final Analysis

The final analysis of all outcomes collectively will take place at the end of trial, which as per the study protocol is defined to be the date on which data for all participants is locked and data entry privileges are withdrawn from the trial database. However, the trial may be closed prematurely by the Trial Steering Committee (TSC), on the recommendation of the IDSMC.

19 Disposition of Participants

A CONSORT flow diagram (Kenneth F Schulz, 2010) will be used to summarise the number of patients who were:

- assessed for eligibility at screening
    - eligible at screening
    - ineligible at screening*
- eligible and randomised
- eligible but not randomised*
- received the randomised allocation
- did not receive the randomised allocation*
- lost to follow-up*
- discontinued the intervention*
- withdrew from follow-up*
- randomised and included in the primary analysis
- randomised and excluded from the primary analysis*

*reasons will be provided.

DRAFT

19.1 Screening, eligibility and recruitment

Screening logs will be summarised by site in a table detailing:

i) the number of patients who were assessed for eligibility at the screening visit,

ii) those who met the study inclusion criteria at screening (expressed as a frequency and a percentage with the denominator being i),

iii) those who did not meet the study inclusion criteria at screening (expressed as a frequency and a percentage with the denominator being i),

iv) those who were eligible from whom consent was obtained, (expressed as a frequency and a percentage with the denominator being all eligible patients ii),

v) those who were eligible from whom consent was not obtained (expressed as a frequency and a percentage with the denominator being all eligible patients ii),

vi) those who provided consent and were randomised (expressed as a frequency and a percentage with the denominator being vi)

vii) those who provided consent but were not randomised (expressed as a frequency and a percentage with the denominator being vi).

Reasons for ineligibility will be summarised overall in a table with the following categories:

1. Not diagnosed mandibular ORN (as specified in protocol Appendix A)
2. Not considered suitable for medical management
3. Cannot swallow tablets
4. Prior treatment with PENTOCLO or any element thereof within 1 year of the date of randomisation
5. Very early ORN (<20 mm^2 exposed bone) occurring within 12 months of a dental extraction or other dentoalveolar operation ('Minor Bone Spicules' see flowchart below)
6. Mandibular pathological fracture secondary to ORN
7. Indication for mandible resection- i.e. patient for whom the severity of their ORN symptoms already constitute an indication for mandible resection and reconstruction. Typically, these symptoms will include severe pain, repeated infections, significant mobile pathological fracture or distressing fistula)
8. Patient has had definitive resection / reconstruction for mandibular ORN – i.e. no longer has exposed necrotic bone present
9. Pregnancy
10. Lactation
11. Age <18 years

12. Acute infection at site of the necrotic bone.[1]
13. Hypersensitivity to other methylxanthines
14. Hypocalcaemia
15. Participants not willing to follow the contraceptive requirements of the protocol
16. Contraindications to PENTOCLO medications:
    a. Known hypersensitivity, allergy or anaphylaxis to pentoxifylline, tocopherol or sodium clodronate
    b. Treated hypotension
    c. Severe coronary artery disease, defined as grade IV of the Canadian Cardiology Society Angina Grading (see Appendix B of the protocol)
    d. Severe cardiac arrhythmia, defined as those cases with attributable syncope or heart failure associated; or those with frequent and symptomatic palpitations, breathlessness, dizziness, chest pain, weakness or fatigue
    e. Myocardial infarction within 6 months
    f. Prior history of extensive retinal haemorrhage
    g. Prior history of intracranial bleeding
    h. Impaired renal function (Creatinine clearance <30 ml/minute, will be formally assessed only if U&E out of reference)
    i. Severe liver failure (class B or C Pugh-Child Score, will be formally assessed only if LFT values out of reference)
    j. Concomitant prescription of anti-platelet agents: clopidogrel, eptifibatide, tirofiban, epoprostenol, iloprost, abciximab, anagrelide, NSAIDs, acetylsalicylates (ASA/LAS) including aspirin >75 mg*, ticlopidine, dipyridamole. (*low dose =<75 mg aspirin is permitted)
    k. Concomitant prescription of ketorolac, cimetidine, ciprofloxacin, theophylline, estramustine phosphate
    l. Hereditary fructose intolerance, glucose-galactose malabsorption or sucrase-isomaltase insufficiency
    m. Concomitant prescription of other bisphosphonates e.g. risedronate, alendronate, albandronate, zoledronic acid, pamidronate, etidronate or prescription of denosusamab
    n. Concomitant prescription of aminoglycoside antibiotics e.g. gentamicin, tobramycin, amikacin, plazomicin, streptomycin, neomycin, paromomycin

Flowchart indicating grade of ORN vs eligibility[(19)]

[1] The acute phase of infection should be controlled by appropriate antibiotics and other measures prior to enrolling the patient into the trial

DRAFT

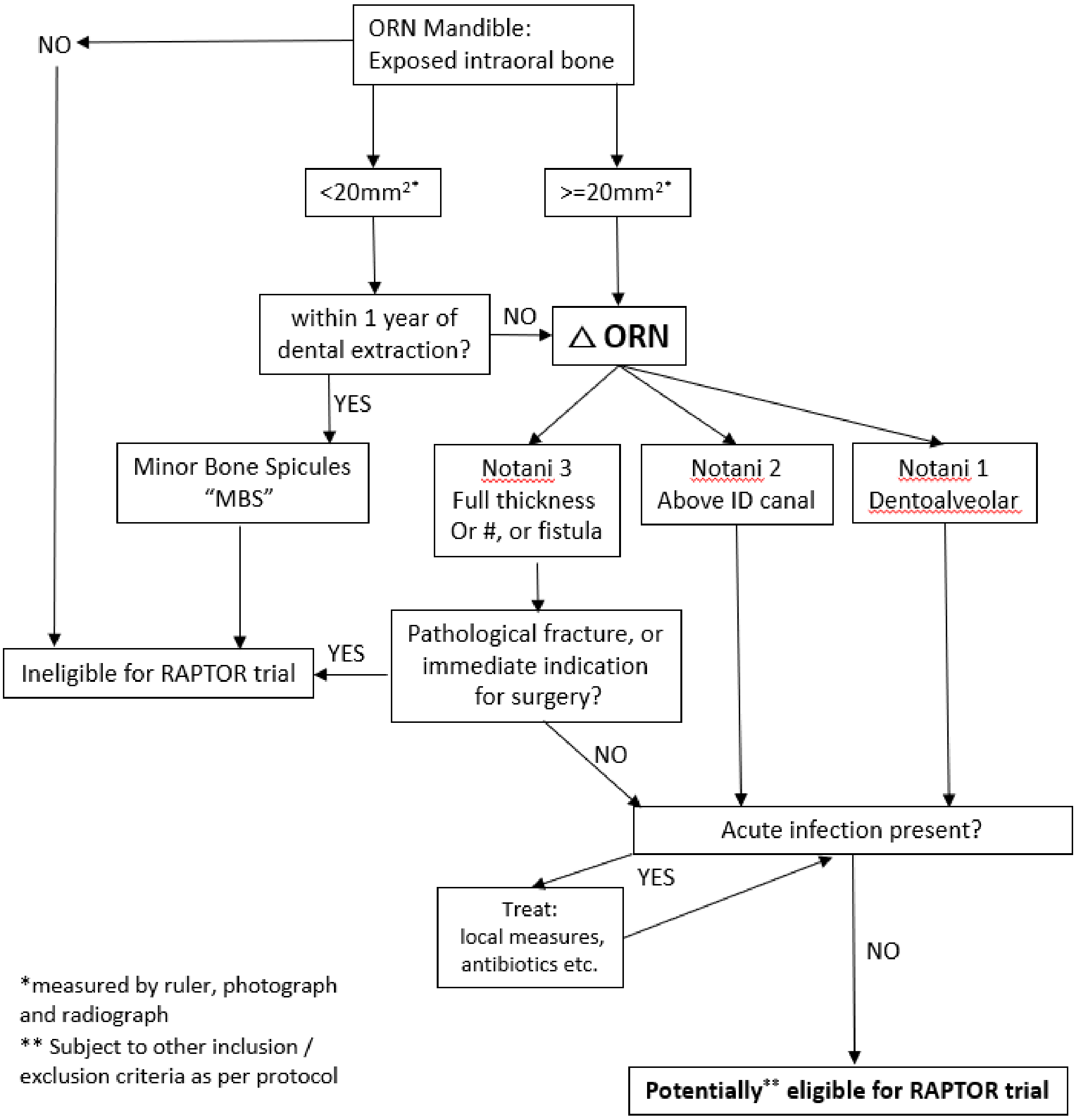

Frequencies will be presented along with percentages using all ineligible patients (ii) as the denominator.

Reasons for declined consent will be summarised overall in a table with the following categories:

a = does not want to take IMP,

b = does not want to take part in research,

c = unwilling to provide a reason

z = other (specify)

Frequencies will be presented along with percentages using all patients where consent was not obtained (vii) as the denominator.

Reasons for consented patients not being randomised will be summarised overall in a table and with the following categories:

1 = No delegated staff available

2 = Other reason (please specify)

Frequencies will be presented along with percentages using all patients who consented but were not randomised (ix) as the denominator

A recruitment summary table will be presented showing the following for each centre: centre code, hospital name, dates site opened/closed to recruitment, dates of first/last randomisation and total number randomised.

19.2 Post randomisation discontinuations

Reasons for participants discontinuing their allocated treatment and patients discontinuing follow up will be summarised where known overall and by treatment arm with the denominator for percentages being the number of participants randomised overall and to that arm as appropriate. The time from randomisation will be recorded. Line listings will be provided for withdrawn participants giving the site, the individual making the decision to withdraw and the reason for withdrawal.

20 Protocol Deviations

Protocol deviations that will be reported are defined in the trial monitoring plan. Protocol deviations are classified prior to release of treatment allocations of treatment to the statistical team. All protocol deviations and patients to be excluded from analysis populations will be defined in one of the following templates: “LCTC_ST003_TEMP4: Protocol Deviations & Analysis Set Definitions for IOC Reports” for Independent Oversight Committee (IOC) reports and “LCTC_ST003_TEMP5: Protocol Deviations & Analysis Set Definitions for Final SARs” for final statistical analysis reports (SAR). These will be signed off by the trial statistician, lead statistician and chief investigator.

For each treatment arm, the number of patients with:

i) each separate protocol deviation;

ii) at least one protocol deviation of any classification (minor or major);

will be presented along with percentages using the number of patients randomised to the treatment arm as the denominator. No formal statistical testing will be undertaken.

21 Unblinding

RAPTOR is an open-label study and thus it is not possible for unblinding to take place.

22 Analysis Datasets

22.1 Efficacy Analyses

The principle of Intention-To-Treat (ITT), as far as practically possible, will be the main strategy of the analysis adopted for the primary outcome and all the secondary outcomes. These analyses will be conducted on all randomised participants, in the treatment arm to which they were allocated, and for whom the outcome(s) of interest have been observed/measured. No imputations will be made.

The membership of the analysis set for each outcome will be determined and documented in LCTC_ST003_TEMP4 and LCTC_ST003_TEMP5 and reasons for participant exclusion will be given prior to the blind being broken and the randomisation lists being requested. Reasons may include missing data, loss to follow up.

The per protocol population will mirror the ITT population but exclude any patients defined as having a major protocol deviation (see section 20). This population will be used in a sensitivity analysis of the primary outcome.

22.2 Safety Analyses

The safety analysis set will include all patients randomised and starting treatment. Safety data will be presented descriptively. Results will be split by treatment arm.

23 Baseline Characteristics

The following baseline characteristics will be summarised for participants overall and by treatment arm:

- Age (years) presented as both a continuous variable and categorical with the categories (18-<65 years, 65-<85 years and >=85 years)
- Sex (Male/Female)

DRAFT

- Ethnicity (White British, Irish, Gypsy or Irish Traveller, Other White, White and Black Caribbean, White and Asian, White and Black African, Other Mixed, Indian, Bangladeshi, Pakistani, Chinese, Black Caribbean, Black African, Black Other, Asian Other, Arab, Other Ethnic Group) and/or a reduced set of categories (White, Mixed/Multiple ethnic group, Asian/Asian British, Black/African/Caribbean/Black British, Other ethnic group)
- BMI (kg/m^2)
- Assessment of medical history
    - Whether or not participants report any medical conditions (categorical)
    - Number of medical conditions reported per participant (continuous)
    - A separate table summarising the number and proportion of participants reporting each MedDRA-coded medical history term will also be presented
- Participants taking the following at baseline:
    - Antibiotics
    - Analgesics
- Blood test results for:
    - U&E
        - Sodium
        - Potassium
        - Urea
        - Creatinine
        - eGFR (if Urea or Creatinine outside of expected range)
    - LFTs
        - Bilirubin
        - ALT
        - AST
        - GGT
- Notani grade of ORN (Grade 1/Grade 2/Grade 3)
- CTCAE v5.0 classification of ORN (Grade 1/Grade 2/Grade 3/Grade 4/Grade 5)
- Extent of exposed bone (mm^2)*

Categorical data will be presented using counts and percentages, continuous data will be presented using number of participants, mean, median, Standard Deviation (SD), minimum, maximum and Inter-quartile range (IQR).

DRAFT

* Extent of exposed bone (mm^2) = length of maximum dimension (mm) × width of dimension perpendicular to maximum (mm). The maximum dimension of exposed bone will be identified and measured. The dimension perpendicular to this will then be measured. These two measurements will be multiplied to estimate area.

24 Compliance with Interventions

See section 25.2.3.10 To evaluate IMP compliance.

25 Analysis of Outcomes

25.1 Interim Reporting

25.1.1 For reports to independent oversight committees

As no formal interim analysis is being undertaken, there will be no formal statistical comparisons between the treatment arms for the primary outcome(s) or any of the secondary outcomes.

25.1.2 Assessment of progression criteria

N/A

25.1.3 Formal Interim Analysis

No formal interim analysis will take place for RAPTOR.

25.2 Final Analysis

25.2.1 Levels of significance and multiplicity

Analysis of the primary outcome will be assessed using 1-tailed 0.1 level as this is consistent with the type I alpha level used in the study design and results will be presented with a one-sided 90% lower bound confidence interval. All analyses of secondary outcomes will use the nominal $p<0.05$ level to determine statistical significance and results will be presented with a 95% confidence interval. No adjustments will be made for multiplicity.

25.2.2 Primary Outcome(s)

To determine if PENTOCLO triple therapy is superior to standard supportive care with respect to time to healing of mandibular ORN.

25.2.2.1 Derivation

This outcome will be derived as time from randomisation to healing of mandibular ORN as determined by clinical examination occurring every 3 months (+/-1 month) following randomisation until study completion. Participants for whom their mandibular ORN has not healed will be censored at the date of their final clinical examination, or if applicable, the date of withdrawal from the study.

Let E be the event: participant has experienced healing of mandibular ORN since randomisation. A participant may either: (a) be interval-censored if they experience the event E at some time point between randomisation and first 3 monthly (+/-1 month) clinical examination or between any two 3 monthly (+/-1 month) clinical examinations; (b) be right-censored before event E can take place; (c) not experience the event during the course of the study and will become right-censored.

Let T0 be the date of randomisation and TE be the date at which they are censored. Then the outcome is defined as T:

$$T = TE - T0$$

where T is measured in days. The date of randomisation (T0) will be taken from the Randomisation CRF as captured in the variable “rand_date”, and the date of clinical examination for which ORN healing was confirmed will also be used to derive TE.

25.2.2.2 Analysis

The interval (in days) from randomisation to healing of mandibular ORN will be summarised by Kaplan-Meier curves for each treatment arm. Comparison between the two treatment groups will be performed using a log rank test. A Cox proportional hazards model will also be fitted with variables included for treatment arm (PENTOCLO vs standard supportive care), Notani grade at baseline and study site. The HR for PENTOCLO vs standard supportive care (both adjusted and unadjusted) will be estimated and presented with a p-value and a one-sided 90% lower bound confidence interval.

The assumption of proportional hazards will be investigated by examining Schoenfeld residual plots, and incorporating time-dependent covariates in all models. If residuals are not time-dependent, and the parameter estimate for the time-dependent covariate is not significant at the 5% level, then the assumption of proportional hazards holds and the overall HR will be presented. Otherwise the extended Cox model with the addition of time-dependent covariates will be used and the time dependent HR will be presented along with the overall HR.

DRAFT

Further to the above, a comparison will be made between data collected on healing via intra-oral examination and assessment of ORN by Notani and CTCAE grade at each 3 monthly (+/- 1 month) clinic visit and clinical photographs taken whenever it has been determined that a participant's mandibular ORN has healed (which may occur at any 3 monthly (+/- 1 month) clinic visit until study completion). It is expected that these photographs will provide further confirmation of healing as determined by clinical examination.

25.2.3 Secondary Outcomes

25.2.3.1 To determine if PENTOCLO is superior to standard supportive care with respect to time from randomisation to worsening of ORN

This outcome is measured at baseline and then every 3 months following randomisation until end of trial for that participant.

25.2.3.1.1 Derivation

This outcome will be derived as time from randomisation to worsening of mandibular ORN as measured by extent of exposed bone measured in two dimensions as mm^2 (maximum dimension of exposed bone (mm) × dimension perpendicular to maximum (mm)) and Notani grade (see Appendix A of protocol) at baseline and at each 3 monthly (+/-1 month) clinical examination following randomisation. Participants will be defined as having worsened if either of the following happens at any time point: (i) extent of exposed bone is greater than it was at baseline; or, (ii) Notani grade is higher than at baseline. The first time point where either of these happens will be considered the point at which the participant worsened. Participants for whom their mandibular ORN has not worsened will be censored at the date of their final clinical examination, or if applicable, the date of withdrawal from the study.

Let E be the event: participant has experienced worsening of mandibular ORN since randomisation. A participant may either: (a) be interval-censored if they experience the event E at some time point between randomisation and first 3 monthly (+/-1 month) clinical examination or between any two 3 monthly (+/-1 month) clinical examinations; (b) be right-censored before event E can take place; (c) not experience the event during the course of the study and will become right-censored.

Let T0 be the date of randomisation and TE be the date at which they are censored. Then the outcome is defined as T:

$$T = TE - T0$$

where T is measured in days. The date of randomisation (T0) will be taken from the Randomisation CRF as captured in the variable “rand_date”, and the date of clinical examination for which ORN worsening was confirmed will also be used to derive TE.

25.2.3.1.2 Analysis

The interval (in days) from randomisation to worsening of mandibular ORN will be summarised by Kaplan-Meier curves for each treatment arm. A Cox proportional hazards model will be fitted with variables included for treatment arm (PENTOCLO vs standard supportive care), Notani grade at baseline and study site. The HR for PENTOCLO vs standard supportive care (both adjusted and unadjusted) will be estimated and presented with a p-value and associated 95% confidence interval.

The assumption of proportional hazards will be investigated by examining Schoenfeld residual plots, and incorporating time-dependent covariates in all models. If residuals are not time-dependent, and the parameter estimate for the time-dependent covariate is not significant at the 5% level, then the assumption of proportional hazards holds and the overall HR will be presented. Otherwise the extended Cox model with the addition of time-dependent covariates will be used and the time dependent HR will be presented along with the overall HR.

25.2.3.2 To compare analgesia use between PENTOCLO and standard supportive care

This outcome is measured every 3 months following randomisation until end of trial for that participant.

25.2.3.2.1 Derivation

This outcome will be derived from the type and dose of any drug taken for pain relief relating to ORN in the 24-hour period leading up to each 3 monthly (+/-1 month) clinic visit. Whether or not a participant has started any new antibiotics or analgesia since the previous visit will be recorded at each 3 monthly (+/-1 month) clinic visit. Data collected for generic drug name and drug indication will be used to identify any drug taken for pain relief. Variables for start date, end date and whether or not treatment is ongoing will then be used to determine whether a participant took any drug for pain relief in the 24-hours prior to clinic visit. A binary categorical variable will be derived as participants taking any pain medication in the 24-hours prior to clinic visit versus those taking none.

25.2.3.2.2 Analysis

Summary statistics will be presented for pain relieving medications taken in the 24-hour period prior to appointment for each clinic visit. These will include frequency and percentage for both type of

DRAFT

medication and dose of medication, as well as mean, SD, median, interquartile range, minimum and maximum for dose of medication. The summary statistics will be presented split by treatment arm.

Further to this, a comparison will be made between those participants taking some pain relief medication relating to ORN versus those taking none at all. A repeated measures, mixed-effects logistic regression model will be fitted for which the dependent variable will be the binary categorical variable for pain medication in the 24-hours prior to clinic visit, as defined in the derivation section above. Fixed effects will be included for the time of clinic visit (3, 6, 9, 12-months), for treatment arm (PENTOCLO vs standard supportive care), and for an interaction between time of clinic visit and treatment arm. Study site effects will be accounted for with random effects. The Odds Ratios (ORs) for PENTOCLO vs standard supportive care (both adjusted for time of clinic visit and unadjusted) will be estimated and presented with p-values and associated 95% confidence intervals. ORs for the interaction between time of clinic visit and treatment arm will determine whether or not a participant receiving PENTOCLO is more or less likely to take some pain medication versus no pain medication in the 24 hours prior to clinic visit, when time of clinic visit increases from 3 months to 6, 9 and 12 months.

25.2.3.3 To compare antibiotic use between PENTOCLO and standard supportive care

This outcome is measured every 3 months following randomisation until end of trial for that participant.

25.2.3.3.1 Derivation

At each 3 monthly (+/-1 month) clinic visit, any antibiotic usage specifically related to ORN occurring since the previous trial visit will be recorded and will include the type of systemic antibiotic, the dose and the number of days for each type.

Participants will be categorised based on their antibiotic use over the full 12-month follow-up period using the following categories:

- 0 antibiotics used: No antibiotic use
- 0 < number of antibiotics used < 2 courses: Mild antibiotic use
- ≥ 2 courses of antibiotics used: Chronic antibiotic use

25.2.3.3.2 Analysis

Summary statistics will be presented for antibiotic usage related to ORN. The number and proportion of participants using antibiotics, as per the categories described above, will be summarised. In

addition, the average number of courses of antibiotics taken will be summarised. The frequency and percentage for both type of antibiotic and dose of antibiotic, as well as mean, SD, median, interquartile range, minimum and maximum for dose of antibiotic will also be presented. The summary statistics will be presented split by treatment arm.

Further to this, an ordinal logistic regression model will be fitted for which the dependent variable will be the ordinal categorical variable of antibiotic use with 3 categories (No antibiotic use, Mild antibiotic use, Chronic antibiotic use). This model will be used to compare antibiotic usage between the two arms. Odds Ratios (ORs) for PENTOCLO vs standard supportive care (both adjusted and unadjusted) will be estimated and presented with p-values and associated 95% confidence intervals. These model results will determine whether or not:

- Participants receiving PENTOCLO and SSC are more or less likely to have "Mild antibiotic use" versus "No antibiotic use" than participants receiving SSC only
- Participants receiving PENTOCLO and SSC are more or less likely to have "Chronic antibiotic use" versus "No antibiotic use" than participants receiving SSC only

25.2.3.4 To compare participants' anthropological measurements between PENTOCLO and standard supportive care

This outcome is measured every 3 months following randomisation until end of trial for that participant.

25.2.3.4.1 Derivation

Participant BMI (kg/m^2) (where kg is a participant's weight in kilograms and m^2 is their height in metres, squared) will be recorded at each 3-monthly clinic visit.

25.2.3.4.2 Analysis

Summary statistics for BMI recorded at baseline and at each 3-monthly (+/- 1 month) visit until study completion (mean, SD, median, interquartile range, minimum and maximum) will be presented split by treatment arm. A plot of BMI over time by treatment arm will also be produced.

A repeated measures, linear mixed-effects regression model will also be fitted with fixed effects included for baseline BMI, the time of clinic visit (3, 6, 9, 12-months), treatment arm (PENTOCLO vs standard supportive care), and an interaction between time of clinic visit and treatment arm. Study site effects will be accounted for with random effects. The adjusted mean difference main effect of PENTOCLO compared to standard supportive care will be estimated and presented with a 95%

confidence interval, along with the corresponding unadjusted effect for completeness. Estimated effects for the interaction between treatment arm and time of clinic visit will also be presented with corresponding 95% confidence intervals.

25.2.3.5 To compare severity of disease between PENTOCLO and standard supportive care measured using CTCAE Grade by "Osteonecrosis of jaw"

This outcome is measured every 3 months following randomisation until end of trial for that participant.

25.2.3.5.1 Derivation

This outcome will be derived using Grade by "Osteonecrosis of jaw" within CTCAE v 5.0 2017 (see Appendix D of protocol - within Musculoskeletal and connective tissue disorders). It will be recorded for each participant at baseline, at each 3 monthly (+/-1 month) intra-oral examination, and at study completion. CTCAE classification of osteonecrosis of the jaw has 5 grades as listed below:

Grade 1: Asymptomatic; clinical or diagnostic observations only, intervention not indicated
Grade 2: Symptomatic; medical intervention indicated (e.g., topical agents); limiting instrumental activities of daily living (ADL)
Grade 3: Severe symptoms; limiting self-care ADL; elective operative intervention indicated
Grade 4: Life-threatening Death consequences; urgent intervention indicated
Grade 5: Death

CTCAE grade will be treated as an ordinal categorical variable.

25.2.3.5.2 Analysis

Summary statistics will be presented for CTCAE grade recorded at baseline, at each 3 monthly (+/-1 month) visit, and at study completion. The frequency and percentage of CTCAE grade will be presented split by treatment arm.

Further to this, an ordinal logistic regression model will be fitted for which the dependent variable will be the ordinal variable CTCAE grade (grade 1, grade 2, grade 3, grade 4, grade 5) and the independent variable will be the treatment allocation (PENTOCLO vs. SSC). This model will be adjusted for baseline CTCAE grade and will be fitted separately for each timepoint (3, 6, 9 and 12 months). Odds ratios will be reported along with associated 95% CIs and p-values.

DRAFT

25.2.3.6 To determine if PENTOCLO is superior to standard supportive care with respect to EORTC QLQ-C30 and QLQ-H&N35 Quality of Life questionnaire scores

This outcome is measured every 3 months following randomisation until end of trial for that participant using the EORTC QLQ-C30 questionnaire with the QLQ-H&N35 module.

25.2.3.6.1 Derivation

The EORTC QLQ-C30 contains five functional scales (physical, role, cognitive, emotional and social), three symptom scales (fatigue, pain, and nausea/vomiting), a global QoL scale, and six single-items (dyspnoea, insomnia, appetite loss, constipation, diarrhoea, and financial difficulties). The QLQ-H&N35 incorporates seven multiple-item scales that assess the symptoms of pain, swallowing, senses (taste/smell), speech, social eating, social contact, and sexuality. Also included are six single-item scales, which survey the presence of symptomatic problems associated with teeth, mouth-opening, dry-mouth (xerostomia), sticky saliva, coughing, and feeling ill. All scales pertaining to the EORTC QLQ-C30 and QLQ-H&N35 range from zero to 100. A high score for a functional or global HR-QoL scale represents a relatively high/healthy level of functioning or global quality of life, whereas a high score for a symptom scale represents the presence of a symptom or problem(s). HR-QoL scales including a global QoL scale will be calculated for each participant at baseline and at 3, 6, 9, and 12 months post-randomisation according to the EORTC QLQ scoring manual, which also covers how missing data will be handled.

25.2.3.6.2 Analysis

Mean scores and standard deviations of the HR-QoL scales will be calculated at baseline and 3, 6, 9, and 12 months following randomisation according to the EORTC QLQ scoring manual and presented split by treatment arm. These scores will also be plotted over time by treatment group.

Further to this, global HR-QoL scale will be analysed using a repeated measures, linear mixed-effects regression model which will include variables for baseline global HR-QoL, the time of clinic visit (3, 6, 9, 12-months), treatment arm (PENTOCLO vs standard supportive care), and an interaction between time of clinic visit and treatment arm. Study site effects will be accounted for with random effects. The adjusted mean difference main effect of PENTOCLO compared to standard supportive care will be estimated and presented with a 95% confidence interval, along with the corresponding unadjusted effect for completeness. Estimated effects for the interaction between treatment arm and time of clinic visit will also be presented with corresponding 95% confidence intervals.

DRAFT

25.2.3.7 To determine if PENTOCLO is superior to standard supportive care with respect to mandibular preservation rate

This outcome is measured every 3 months following randomisation until end of trial for that participant.

25.2.3.7.1 Derivation

Whether or not a participant has had jaw resection surgery since the previous visit is to be recorded at each 3 monthly (+/- 1 month) clinic visit until the end of trial. They type of jaw resection surgery (marginal or segmental) will also be recorded. From this, the mandibular preservation rate will be derived as the number of participants who have not undergone surgery to remove the mandible (segmental resection, with or without reconstruction) at 3, 6, 9, 12-months and study completion.

Further to the preservation rate, the time from randomisation to surgery to remove the mandible will be derived. Participants who have not undergone surgery to remove the mandible will be censored at the date of their final clinical examination, or if applicable, the date of withdrawal from the study.

Let E be the event: participant has undergone surgery to remove the mandible since randomisation. A participant may either: (a) be interval-censored if they experience the event E at some time point between randomisation and first 3 monthly (+/-1 month) clinical examination or between any two 3 monthly (+/-1 month) clinical examinations; (b) be right-censored before event E can take place; (c) not experience the event during the course of the study and will become right-censored.

Let T0 be the date of randomisation and TE be the date at which they are censored. Then the outcome is defined as T:

$$T = TE - T0$$

where T is measured in days. The date of randomisation (T0) will be taken from the Randomisation CRF as captured in the variable “rand_date”, and the date of clinical examination for which ORN worsening was confirmed will also be used to derive TE.

25.2.3.7.2 Analysis

A summary of the mandibular preservation rate at 3, 6, 9, 12-months and study completion will be presented split by treatment arm and overall. The mandibular preservation rate will also be analysed using a repeated measures, mixed-effects logistic regression model with variables include for study site, a categorical variable for time of clinic visit (3, 6, 9, 12-months) and treatment arm (PENTOCLO vs standard supportive care). Study site effects will be accounted for with random effects. The Odds

Ratios (ORs) for PENTOCLO vs standard supportive care (both adjusted for time of clinic visit and unadjusted) will be estimated and presented with p-values and associated 95% confidence intervals.

Further to the above, the interval (in days) from randomisation to surgery to remove the mandible will be summarised by Kaplan-Meier curves for each treatment arm. A Cox proportional hazards model will be fitted with variables included for treatment arm (PENTOCLO vs standard supportive care), Notani grade at baseline and study site. The Hazard Ratio (HR) for PENTOCLO vs standard supportive care (both adjusted and unadjusted) will be estimated and presented with a p-value and associated 95% confidence interval.

The assumption of proportional hazards will be investigated by examining Schoenfeld residual plots, and incorporating time-dependent covariates in all models. If residuals are not time-dependent, and the parameter estimate for the time-dependent covariate is not significant at the 5% level, then the assumption of proportional hazards holds and the overall HR will be presented. Otherwise the extended Cox model with the addition of time-dependent covariates will be used and the time dependent HR will be presented along with the overall HR.

25.2.3.8 To determine gastrointestinal tolerability of PENTOCLO regimen as defined by CTCAE grading

This outcome is measured 2 weeks (+/- 5 working days) after commencing trial medications and only for participants in Arm B: SSC and PENTOCLO

25.2.3.8.1 Derivation

This outcome is derived as the number of and grade of gastrointestinal adverse events (as defined by CTCAE grading) experienced during the first two weeks following commencement of trial medication by participants in Arm B: SSC and PENTOCLO.

25.2.3.8.2 Analysis

Line listings will be reported for gastrointestinal adverse events occurring first two weeks following commencement of trial medication by participants in Arm B: SSC and PENTOCLO.

A summary of the total number and percentage of participants experiencing at least one of these events will be presented, as well as a summary of the total number of events experienced per participant. This will also be presented for individual events which occur more than once.

25.2.3.9 To evaluate IMP compliance

This outcome is measured every 3 months following randomisation until the end of trial for that participant and only for participants in Arm B: SSC and PENTOCLO.

25.2.3.9.1 Derivation

Whether or not a participant is still taking PENTOCLO will be recorded at each 3 monthly (+/-1 month) clinic visit. If the answer to this question is no, then a premature discontinuation form will be completed. This will capture the date of the discontinuation and the reason for discontinuation, which may be given as one of the following reasons:

1. Participant led, please specify
2. Unacceptable toxicity
3. Intercurrent illness preventing further treatment
4. Pregnancy
5. Death
6. Clinician-led please specify
7. Withdrawal from study
8. Other, please specify

Using the data collected as described above, instances of non-compliance will be identified via the reasons specified for any recorded “Participant led, please specify” or “Clinician-led please specify” decision to discontinue treatment of PENTOCLO and potentially, “Other, please specify” reasons given for premature discontinuation too. The dates recorded for premature discontinuation for any identified instances of non-compliance will be used to calculate the number of days of compliance and non-compliance as at 3, 6, 9, and 12-months.

25.2.3.9.2 Analysis

The number and percentage of participants in the PENTOCLO arm with non-compliance at 3, 6, 9, and 12-months will be presented. Summary statistics will be provided for the number of days of compliance and non-compliance, which will include the total, mean, SD, median, interquartile range, minimum and maximum number of days. Frequency and percentages for reasons provided for non-compliance for participants in the PENTOCLO arm will also be presented.

DRAFT

25.2.3.10 To evaluate IMP's combination safety

This outcome is measured every 3 months following randomisation until the end of trial for that participant and only for participants in Arm B: SSC and PENTOCLO.

25.2.3.10.1 Derivation

This outcome is derived as the number of and grade of severe adverse events (severe AEs) and/or serious adverse events (SAEs), considered related to the study treatment, experienced during the trial.

25.2.3.10.2 Analysis

Line listings will be reported for severe adverse events (severe AEs) and/or serious adverse events (SAEs), considered related to the study treatment, experienced during the trial by participants in Arm B: SSC and PENTOCLO.

A summary of the total number of severe AEs and SAEs considered related to the study treatment reported for participants in the PENTOCLO arm will be presented, along with the number of participants with experiencing at least 1 severe AE and/or SAE and summary statistics for the number of severe AEs/SAEs considered related to the study treatment reported per participant (mean, SD, median, interquartile range, minimum and maximum).

Further to this, a summary of all AEs and ARs reported throughout the trial for all participants will be presented split by treatment arm. This is covered in SAP section 26 Safety Evaluations.

25.2.4 Exploratory outcomes

25.2.4.1 Investigation of self-reported participant measures data gathered via the eProm app

This outcome comprises participants' self-reported pain medication use in the previous 24 hours (including type and dose of medication), four self-reported participant measures relating to pain and mouth function i.e., pain, eating, mouth-opening, and problems with teeth/gums, details of painkillers taken in the last 24 hours and six self-reported participant measures relating to interference of symptoms with participants' daily activities i.e., general activity, mood, work (including work around the house), relations with other people, walking, and enjoyment of life and is collected every 15 days until end of trial for the participant via the ePROM app. The key purpose of this exploratory outcome is to understand whether it is possible to collect data in this way.

25.2.4.1.1 Derivation

Every 15 days and for each of the measures relating to pain and mouth function, participants are asked to rate the severity of the issue on a scale of 0 – 10 for which 0 represents "NOT PRESENT" and 10 represents "AS BAD AS YOU CAN IMAGINE". The full questions, as asked via the ePROM app, for these four measures are as follows:

- Please rate the severity of your pain at its WORST in the last 24 hours?
- Please rate the severity of your difficulty swallowing/chewing at its WORST in the last 24 hours?
- Please rate the severity of any problem with your teeth or gums at its WORST in the last 24 hours?
- Please rate the severity of any difficulty with opening your mouth at its WORST in the last 24 hours?

Every 15 days and for each of the measures relating to interference of symptoms with participants' daily activities, participants are asked to rate how much symptoms interfered on a scale of 0 – 10 for which 0 represents "Did not interfere" and 10 represents "Interfered Completely". The full questions, as asked via the ePROM app, for these six measures are as follows:

- How much have your symptoms interfered with general activity in the last 24 hours?
- How much have your symptoms interfered with mood in the last 24 hours?
- How much have your symptoms interfered with work (including work around the house) in the last 24 hours?
- How much have your symptoms interfered with relations with other people in the last 24 hours?
- How much have your symptoms interfered with walking in the last 24 hours?
- How much have your symptoms interfered with enjoyment of life in the last 24 hours?

Every 15 days, the ePROM app also asks participants whether they have taken any pain relief medication in the last 24 hours and if they answer yes to this, they are asked to provide the type of pain relief medication, the dose, and the number of times taken in the previous 24 hours.

25.2.4.1.2 Analysis

The primary interest relating to this outcome is the rate of completion. A summary of data completeness gathered via the ePROM app for at each 15-day post-randomisation period individually and cumulatively until study completion will be presented for the study overall and split by treatment arm. This will include the frequency and percentage of completed ePROM questionnaires.

DRAFT

Further to this, summary statistics will be presented for each measure relating to pain and mouth function and for each measure relating to interference of symptoms in the previous 24-hours. These will include mean, SD, median, interquartile range, minimum and maximum score for each measure. Summary statistics will also be presented for pain relieving medications taken in the previous 24-hours. These will include frequency and percentage of participants taking painkillers in the previous 24 hours. The summary statistics will be presented split by treatment arm.

26 Safety Evaluations

For RAPTOR, the safety analysis data set will include all participants randomised and starting treatment. Safety data will be presented descriptively. Results will be split by treatment arm. Line listings will be provided for SAEs, detailing the description, seriousness, severity and expectedness. All non-serious AEs and SAEs reported by the clinical investigator will be presented in a table. The number (and percentage) of patients experiencing each AE/SAE will be presented for each treatment arm categorised by severity. For each patient, only the maximum severity experienced of each type of AE will be displayed. The number (and percentage) of occurrences of each AE/SAE will also be presented for each treatment arm. No formal statistical testing will be undertaken. Safety data will be quality-checked by a statistician otherwise not involved in the RAPTOR trial.

27 Additional Analyses

N/A.

DRAFT

28 Document History

Statistical Analysis Plan Version	Protocol Version	Section number(s) changed	Description of changes	Justification for changes	Date Implemented

DRAFT

29 References

29.1 Non-standard statistical methods

N/A.

29.2 Data Management Plan

RAPTOR Data Management Plan V1.0 (03/04/2023)

29.3 Trial Master File and Trial Statistical File

RAPTOR Trial Master File

RAPTOR Trial Statistical File

29.4 Other Standard Operating Procedures to be adhered to

LCTC_ST003: Production of Statistical Analysis Plans and Reports

LCTC_ST004: Quality Control of Statistical Programming

LCTC_ST005: Statistical Programmed Data Checks

LCTC_ST006: Creating and Maintaining a Trial Statistical File

29.5 Other references

1. Harris M. The conservative management of osteoradionecrosis of the mandible with ultrasound therapy. Br J Oral Maxillofac Surg. 1992;30(5):313-8.
2. Rice N, Polyzois I, Ekanayake K, Omer O, Stassen LF. The management of osteoradionecrosis of the jaws--a review. Surgeon. 2015;13(2):101-9.
3. Shaw RJ, Butterworth CJ, Silcocks P, Tesfaye BT, Bickerstaff M, Jackson R, et al. HOPON (Hyperbaric Oxygen for the Prevention of Osteoradionecrosis): A Randomized Controlled Trial of Hyperbaric Oxygen to Prevent Osteoradionecrosis of the Irradiated Mandible After Dentoalveolar Surgery. Int J Radiat Oncol Biol Phys. 2019;104(3):530-9.
4. Annane D, Depondt J, Aubert P, Villart M, Gehanno P, Gajdos P, et al. Hyperbaric oxygen therapy for radionecrosis of the jaw: a randomized, placebo-controlled, double-blind trial from the ORN96 study group. J Clin Oncol. 2004;22(24):4893-900.
5. Dhanda J, Rennie L, Shaw R. Current trends in the medical management of osteoradionecrosis using triple therapy. Br J Oral Maxillofac Surg. 2018;56(5):401-5.

DRAFT
