# Supplementary material for: RAPTOR: Randomised Controlled Trial of PENTOCLO (pentoxifylline-tocopherol-clodronate) in Mandibular Osteoradionecrosis—study protocol for an open-label phase II randomised controlled superiority trial
Source: Trials. 2025 Jul 24;26:254. doi: 10.1186/s13063-025-08966-9 (PMC12291413; doi:10.1186/s13063-025-08966-9)
Supplement: Supplementary file 2 — Supplementary Material 2: Appendix 2. Patient Information and Consent RAPTOR Trial [file 13063_2025_8966_MOESM2_ESM.docx]

- You have been invited to take part in a research study. Before you decide whether to take part in this study, it is important that you understand why the research is being done and what it will involve.
- Please take time to read the following information carefully. **Part 1** tells you the purpose of the study and what will happen to you if you take part. **Part 2** gives you more detailed information about the conduct of the study.

| **Contents** | |
| --- | --- |
| **Part 1** | **2** |
| Why are we doing the RAPTOR study? | 2 |
| Why have I been chosen? | 2 |
| Do I have to take part? | 2 |
| What will happen to me if I take part? | 2 |
| Study Timeline | 3 |
| Study Assessments Table | 4 |
| What is the drug being tested? | 5 |
| How will I know which treatment I’m going to have? | 5 |
| What are the alternatives for treatment? | 5 |
| What are the benefits and risks of taking part? | 5 |
| What happens if I change my mind? | 6 |
| What if new information becomes available? | 6 |
| What happens when the study stops? | 6 |
| What if there is a problem? | 6 |
| Will my taking part in the study be kept confidential? | 6 |
| **Part 2** | **7** |
| Who is running the study? | 7 |
| How will my information be collected and handled? | 7 |
| What are my choices about how my information is used? | 7 |
| Information sharing for other research | 7 |
| Where can I find out more about how my information is used? | 8 |
| What will happen to the blood samples I give? | 8 |
| What if there’s a problem? | 8 |

- You can ask a member of your clinical team (doctor, nurse) if there is anything that is not clear, or if you would like more information.
- If you wish you can discuss it with friends, relatives and/or get independent advice via your local Patient Advice and Liaison Service (PALS) or equivalent. [Members of your local hospital team should be able to provide these contact details to you.]
- Taking part is voluntary. If you don’t want to take part, then please don’t worry. There is no need to give a reason for not participating and you will, of course, receive the usual treatment the hospital offers for your condition.
- RAPTOR is a study running across the UK, which aims to recruit 120 patients that have a damaged jaw caused by radiotherapy treatment, called osteoradionecrosis.
- The RAPTOR trial acronym stands for ‘Randomised Controlled Trial of Pentoxifylline, Tocopherol & Clodronate in Mandibular Osteoradionecrosis’
- Pentoxifylline, Tocopherol and Clodronate are tablets which will be taken daily for at least 1 year.
- Participants will be enrolled in the study for up to 3 years to ensure that the full long-term impact of each treatment can be analysed and assessed.

| **How to contact the local study team** |
| --- |
| If you have any questions about this study, please talk to your research team:  **Principal Investigator:** <PI Name>  **Research Nurse:** <RN Name>  **Telephone:** <Number>  **Or visit the website:** <https://raptorstudy.org.uk/> |

PART 1: Purpose of the study and what will happen if you take part

Why are we doing the RAPTOR study?

Osteoradionecrosis is a complication of radiotherapy following the treatment of head and neck cancer. It is the death of the jawbone caused by irradiation, which leads to infection, pain, and fracture. Patients with osteoradionecrosis can experience difficulties with swallowing, chewing, speech, which can even lead to weight loss and changes in facial appearance. It is estimated that one in fifteen patients treated for head and neck cancer with radiotherapy will develop osteoradionecrosis.

In routine supportive care, osteoradionecrosis is usually treated with painkillers, antibiotics and mouthwashes to control infection, and removal of any sharp or loose bone. In more serious cases, surgery is required to remove all damaged bone and reconstruct the jawbone, which is a very complex major procedure.

PENTOCLO is a combination of Pentoxifylline, Tocopherol, and Clodronate, which are thought to heal the damage caused by radiotherapy. The potential benefits of PENTOCLO are not yet known, which is why we are doing this research. At present we believe at least 12 months of treatment with PENTOCLO is required to see any benefit.

The RAPTOR trial aims to compare standard treatment against standard treatment with the addition of PENTOCLO. The study will recruit 120 patients from at least 12 different hospitals from around the UK.

Participants will be given treatment for at least 1 year and followed up for a maximum of 3 years.

The results from this study will be used to help us improve treatments for patients with osteoradionecrosis.

Why have I been chosen?

You have been invited to take part in this study because you have osteoradionecrosis of the lower jaw.

Do I have to take part?

No, taking part is voluntary. It is up to you to decide whether or not you want to take part.

If you decide not to take part, then you will still receive the usual treatment your hospital offers. Your doctor can provide you with more information about this.

If you decide to take part, you can also choose to stop at any time without giving a reason.

The decision you make on whether to take part or not will not affect the standard of care you receive now or in the future.

What will happen to me if I take part?

If you agree to take part, you will be asked to sign the consent form at the end of this document. You will be given a copy of the consent form and this information sheet to keep.

Once you have signed the consent form, we will check and confirm that this study is suitable for you, and you will be asked to follow the study plan (see study timeline).

At the start of the study, we will need to take some blood tests to assess your liver and kidney function, check your medical history and any current medications, physical examination of your head and neck and including looking into your mouth, height and weight, take some X-rays and photographs of your jaw and complete baseline questionnaires. If X-rays have already been taken as part of your usual care, we will use these images instead.

If you are a person of childbearing potential, we will also do a blood test to check whether or not you are pregnant.

When you are entered into the study, you will randomly be allocated to either a standard treatment or standard treatment and PENTOCLO. If you are allocated to PENTOCLO, you will need to take tablets every day for at least one year.

All participants will need to attend hospital visits every three months for at least one year, for a maximum of three years or until the jaw is confirmed to be healing. These visits are considered to be in line with your routine care. At each of these visits, we will repeat the assessments taken at the start of the study.


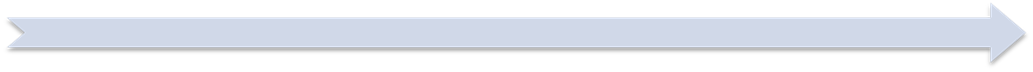

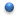

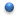

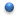

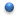


Within 8 weeks of Baseline


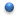


Every 15 days

Month 12 and every 3 months for participants that continue beyond 12 months


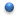


1-3 weeks after Randomisation


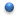


Months 3, 6, and 9

**Joining RAPTOR**

Consent

**Baseline**

Blood tests, medication and medical history check, physical examination, ECG, X-ray, and photographs of the jaw.

Questionnaires.

**Randomisation**

You will be allocated to receive either Standard care or PENTOCLO + Standard care.

Week 0

Week 0

**Clinic Visits 2**

Review of medication and medical history, physical examination.

X-ray and photograph of the jaw at 12 months and study completion.

Questionnaires.

Arm B only; blood tests, ECG*

*these tests will be done every 6 months until study completion.

**Clinic Visits 1**

Review of medication and medical history, physical examination.

Questionnaires.

Arm B only; blood tests, ECG*

*these tests will only be done on 3 and 6 monthly visits.

**App completion**

  Questions should be completed via the smartphone app, online or telephone.

**Telephone Check-up**

  The study team will call to check how you are getting on with the treatment and using the App

After one year, you can decide to end the trial or to continue for up to three years, this is at your preference.

At your clinic visits, we ask that you complete a set of questionnaires. These questionnaires will ask about your symptoms and quality of life.

We will also have brief questions about symptoms that can be completed via a smartphone App. We will demonstrate how to use the App and the App will prompt you to report any symptoms you may have every 15 days. This should only take a minute or so. If you don’t use a smartphone we can also collect this data online, or via telephone.

| **When** | **Procedure** | **Description** | **Research Treatment or**  **Standard of care** |
| --- | --- | --- | --- |
| Screening and Baseline | Informed Consent | You will be given time to read and sign the consent form and ask any questions. | ***Research Treatment*** |
|  | Blood Tests | Full blood count, clotting and calcium level tests  Liver function tests (LFT) and Urea & electrolytes (UE) are used to assess liver and renal function.  Follicle Stimulating Hormone (FSH) test to confirm postmenopausal state and pregnancy test if you are a person of childbearing potential. | **Standard of Care** |
|  |  | Collection of blood samples for future research | ***Research Treatment*** |
|  | Radiograph | An orthopantomogram (jaw X-Ray) is required to define the stage of osteoradionecrosis. | **Standard of Care** |
|  | Medical Assessment and History | Assessment of medical history.  List of current medications.  Height & Weight. | **Standard of Care** |
|  | Physical examination | General oral/head and neck examination.  Intra-oral examination and assessment of osteoradionecrosis.  Clinical photograph of osteoradionecrosis. | **Standard of Care** |
|  | ECG | To monitor the cardiac health | ***Research Treatment*** |
|  | Questionnaires | App activation and training. | ***Research Treatment*** |
|  |  | Completion of EORTC QLQ-C30 and QLQ-H&N35 |  |
| Within 8 weeks of first baseline assessment | Randomisation | You will be randomly allocated to receive either:   - Standard Care, or - PENTOCLO + Standard Care | ***Research Treatment*** |
| Every 15 days until end of participation | Questionnaires | Complete Smartphone App data (or online/by telephone) | ***Research Treatment*** |
| 2 weeks after randomisation | Telephone Check | To check how you are getting on with treatment and completing the App data | ***Research Treatment*** |
| Months 3, 6, and 9 | Blood Tests | Full blood count, clotting and calcium level tests  Liver function tests (LFT) and Urea & electrolytes (UE) are used to assess liver and renal function.  Pregnancy test only if you are a person of childbearing potential. | ***Research Treatment (Arm B only)*** |
|  | Medical Assessment and History | Assessment of medical history.  List of current medications.  Height & Weight. | **Standard of Care** |
|  | Physical examination | General oral/head and neck examination.  Intra-oral examination and assessment of osteoradionecrosis.  Clinical photograph of osteoradionecrosis. | **Standard of Care** |
|  | ECG | To monitor the cardiac health | ***Research Treatment (Arm B only)*** |
|  | Questionnaires | Completion of EORTC QLQ-C30 and QLQ-H&N35. | ***Research Treatment*** |
| Months 12 and then every 3 months for participants that continue beyond 12 months, until 3 years. | Blood Tests | Full blood count, clotting and calcium level tests  Liver function tests (LFT) and Urea & electrolytes (UE) are used to assess liver and renal function.  Pregnancy test only if you are a person of childbearing potential. | ***Research Treatment (Arm B only)*** |
|  | Medical Assessment and History | Assessment of medical history.  List of current medications.  Height & Weight. | **Standard of Care** |
|  | Physical examination | General oral/head and neck examination.  Intra-oral examination and assessment of osteoradionecrosis. | **Standard of Care** |
|  | ECG | To monitor the cardiac health | ***Research Treatment (Arm B only)*** |
|  | Questionnaires | Completion of EORTC QLQ-C30 and QLQ-H&N35 | ***Research Treatment*** |
|  | Radiograph | An orthopantomogram (jaw X-Ray) is required to define the stage of osteoradionecrosis. | **Standard of Care** |

What is the drug being tested?

PENTOCLO is a combination of Pentoxifylline, Tocopherol and Clodronate, which are tablets that are to be taken orally.

**Pentoxifylline**: 1 tablet (400 mg) twice daily, taken with or immediately after meals, and swallowed whole with plenty of water.

**Tocopherol (Vitamin E):** Suspension 1000 mg once daily.

**Sodium Clodronate**: 1600 mg capsules should be taken as a single dose, but only Monday to Friday, i.e. excluding Saturday and Sunday. This should preferably be taken in the morning on an empty stomach together with a glass of water, then refrain from eating, drinking (other than plain water), and taking any other oral drugs for one hour. Alternatively, between meals, more than two hours after and one hour before eating, drinking (other than plain water).

How will I know which treatment I’m going to have?

In research studies we often split patients up into groups to look at how different treatments work. In the RAPTOR study patients will be split into two treatment groups at random:

- One group will receive Standard Care
- The other group will receive PENTOCLO and Standard Care

It is really important that each group in the RAPTOR study has a similar mix of patients in it, so we know that if one group of patients does better than the other it is very likely to be because of the treatment and not because there are differences in the types of patients in each group.

We use a computer programme that puts patients into groups ‘at random’ – you might hear this described as ‘randomisation’ or ‘random allocation’, but they all mean the same thing. Neither you nor your doctor choose which group you are in.

In the RAPTOR study you are equally as likely to be in the group receiving Standard Care as you are in the group receiving PENTOCLO.

Your healthcare team will let you know which group you are in as soon as possible.

What are the alternatives for treatment?

In standard care, painkillers, antibiotics and mouthwashes to control infection, and minor procedures to remove any sharp or loose bone are the usual treatments to manage osteoradionecrosis.

In more serious cases, surgery is sometimes required to remove all damaged bone and reconstruct the jawbone, which can be a complex, major procedure.

If your jaw osteoradionecrosis did get worse, you might be offered surgical removal of the dead bone and reconstruction. This could happen with either treatment, either standard care or PENTOCLO. As part of the trial, you will still receive any surgery that you need, but it would mark the end of your trial participation.

What are the benefits and risks of taking part?

Both PENTOCLO and Standard Care have been shown to improve symptoms, but this cannot be guaranteed. We also do not know which treatment is best.

With standard care, you will be given painkillers, antiseptic mouthwash, antibiotics (sometimes intermittent, sometimes prolonged course), occasionally antifungal agents and steroids. Each of these measures carries its own element of risk and side effects which your doctor or nurse will discuss with you.

Experience from previous patients has shown that they can usually take PENTOCLO without serious side effects. Possible side effects of PENTOCLO medications include: dizziness and headache, abdominal discomfort, nausea, vomiting and diarrhoea. If you experience these, the doses can be reduced to lessen these symptoms. If the symptom persisted despite a reduction, you could stop the medications if needed and continue standard care.

Blood samples will be collected during the study, which may result in mild pain, bruising or redness at the needle site. These will be minor and should clear-up after a few days.

If you take part in this study you will undergo panoramic dental X-rays of your jaw. Some of these procedures may be extra to those that you would have if you did not take part. These procedures use ionising radiation to form images of your body and provide your doctor with other clinical information. Ionising radiation can cause cell damage that may, after many years or decades, turn cancerous.

We are all at risk of developing cancer during our lifetime. 50% of the population is likely to develop one of the many forms of cancer at some stage during our lifetime. Taking part in this study will add only a very small chance of this happening to you.

We hope that the results from the study will help doctors and patients in the future when making decisions about treatment.

What happens if I change my mind?

If at any point you decide to stop taking part in the study, you will still receive the standard treatment and the follow up usually offered by your hospital.

If you do decide to stop taking part, we will ask you if you would like to:

- continue to complete follow up visits for the study **or**
- stop taking part with no more study visits.

Information on how we will handle your information and samples in the event of you withdrawing is detailed in Part 2 of this Information Sheet.

What if new information becomes available?

Sometimes during the course of a research project, important new information becomes available about the treatment/drug that is being studied. If this happens, your doctor will tell you about it and discuss with you whether you want to or should continue in the study. If you decide to withdraw your doctor will make arrangements for your care to continue. If you decide to continue in the study, you will be asked to sign an updated consent form.

On receiving new information your doctor might consider it to be in your best interests to withdraw you from the study. He/she will explain the reasons and arrange for your care to continue.

If the study is stopped for any other reason, you will be told why, and your continuing care will be arranged.

 What happens when the study stops?

At the end of the study, you will continue to receive the usual treatment your hospital offers. Your doctor will be able to give you more information on the options available.

It is intended that the results of the study will be presented at conferences and published in medical journals so that we can explain to the medical community what our research results have shown. They may also be used to apply to the regulatory authorities to make the drug widely available and/or for research related to the development of pharmaceutical products, diagnostics or medical aids. Confidentiality will be ensured at all times, and you will not be identified in any publication.

Any information derived directly or indirectly from this research, as well as any patents, diagnostic tests, drugs, or biological products developed directly or indirectly as a result of this research may be used for commercial purposes. You have no right to this property or to any share of the profits that may be earned directly or indirectly as a result of this research. However, in signing this form and donating tissue and/or blood samples for this research, you do not give up any rights that you would otherwise have as a participant in research.

What if there is a problem?

Any complaint about the way you have been dealt with during the study or any possible harm you might suffer will be addressed. Detailed information is given in Part 2 of this information sheet.

Will my taking part in the study be kept confidential?

Yes. All the confidential information about your participation in this study will be kept confidential. Detailed information on this is given in Part 2.

PART 2: Detailed information about the conduct of the study

Who is running the study?

The University of Liverpool is the Sponsor of this study and is responsible for managing it. They are based in United Kingdom. They have asked that the day to day running of the study is carried out by a team based at the Liverpool Clinical Trials Centre (LCTC, part of the University of Liverpool).

The study has been reviewed by the Medicines and Healthcare Products (MHRA) Regulatory Agency, the Health Research Authority and the National Research Ethics Service Committee to make sure that the study is scientifically and ethically acceptable.

This study is funded by the National Institute for Health Research (NIHR) Efficacy and Mechanism Evaluation (EME) Programme.

Your doctor will not receive any personal payment for including you in this study. The hospital may receive additional funding to help with any extra costs that supporting this study might incur.

How will my information be collected and handled?

The University of Liverpool is the Data Controller for this study and will need to use information from you and your medical records for this research project.

This information will include your:

- Initials
- Name
- Age

People will use this information to do the research or to check your records to make sure that the research is being done properly.

Individuals from the University of Liverpool, the LCTC and regulatory organisations may look at your medical and research records to check the accuracy of the research study.

People who do not need to know who you are will not be able to see your name or contact details. Your data will have a code number instead.

- main data from site 🡪 LCTC
- blood sample data from site 🡪 University of Liverpool GCP Lab Facility

Data will be sent from your hospital to the LCTC.

We will notify your GP that you will be taking part in the study for their information.

We will keep all information about you safe and secure.

A copy of your completed consent form will be kept in Liverpool Clinical Trials Centre [LCTC] and Liverpool GCP Lab Facility (where it will be stored in a secure location) to allow confirmation that the consent was given. We retain the consent form on their behalf for the storage of samples, they do not receive a copy.

Once we have finished the study, we will keep the data for 25 years, so we can check the results. We will write our reports in a way that no-one can work out that you took part in the study.

What are my choices about how my information is used?

You can stop being part of the study at any time, without giving a reason, but we will keep information about you that we already have.

If you choose to stop taking part in the study, we would like to continue collecting information about your health from your hospital. If you do not want this to happen, tell us and we will stop.

In some cases, however we may need to continue to collect limited information about any side-effects of the study treatment you may experience or pregnancies. We will only do this where we are required to do so by law.

We need to manage your records in specific ways for the research to be reliable. This means that we won’t be able to let you see or change the data we hold about you.

Information sharing for other research

When you agree to take part in a research study, the information about your health and care may be beneficial to researchers running other research studies in this organisation and in other organisations. These organisations may be universities, NHS organisations or companies involved in health and care research in this country or abroad. Your information will only be used by organisations and researchers to conduct research in accordance with the [UK Policy Framework for Health and Social Care Research](https://www.hra.nhs.uk/planning-and-improving-research/policies-standards-legislation/uk-policy-framework-health-social-care-research/), or equivalent standards**.**

If you agree to take part in this study, you will have the option to take part in future research using your data saved from this study.

Where can I find out more about how my information is used?

You can find out more about how we use your information:

- at [www.hra.nhs.uk/information-about-patients](http://www.hra.nhs.uk/information-about-patients)
- by asking one of the research team
- by sending an email to <site **email address**> or
- by calling us on <site **phone number**>
- in the Health Research Authority leaflet available from [www.hra.nhs.uk/patientdataandresearch](http://www.hra.nhs.uk/patientdataandresearch)
- by contacting the University of Liverpool Data Protection Officer on [LegalServices@liverpool.ac.uk](mailto:LegalServices@liverpool.ac.uk)
- In the LCTC’s “Privacy Notice” available from: <https://www.lctc.org.uk/privacy>

If you are not happy with the way your information is being handled, or with the response received from us, you have the right to lodge a complaint with the Information Commissioner’s Office at Wycliffe House, Water Lane, Wilmslow, SK9 5AF ([www.ico.org.uk](https://lctc.org.uk/www.ico.org.uk)).

What will happen to the blood samples I give?

Some of your samples will be analysed at your hospital for the purpose of your treatment.

With your permission, we would also like to collect an extra sample of blood to be used in future research. This would be a single sample of 10 ml, around 2 teaspoons. If you agree, coded samples will be sent to the University of Liverpool GCP Lab Facility for storage. Some of the data we collect about you in this study will be provided alongside your samples – this too will be coded. Coded is not the same as anonymous. It will be possible to use the codes to identify that a result is from your sample. These researchers work closely with other scientists in the UK and elsewhere outside of the UK and may transfer your samples to these research collaborators for use in future scientific studies..

These samples may be used for research to understand if there are genetic causes of osteoradionecrosis.

The sample will be kept in a secure place until we need it and may be retained indefinitely; nobody outside of the study will have access to **any** confidential information that you give to us. Confidential details (such as your name, address and GP details) will be kept locally and not made available to collaborators.

If you choose to withdraw from the study, we will continue to store the samples we have already collected, and it will be made available to future researchers. If you do not want us to do this, please let us know and we will stop where possible. It may however not be possible to withdraw your sample if it has already been included in research.

What if there is a problem?

If you have a concern about any aspect of this study, you should ask to speak with one of your research team who will do their best to answer your questions.

If you remain unhappy and wish to complain formally, you can do this by contacting local NHS Patient Advice and Liaison Service (PALS) or equivalent. Members of your local hospital team should be able to provide this information to you.

Every care will be taken in the course of this clinical study. However, in the very unlikely event that you are harmed by taking part in this research project of the study Sponsor (University of Liverpool), compensation may be available, and you may have to pay your related legal costs. University of Liverpool provides cover for no fault claims.

Your hospital where you receive your treatment has a duty of care to you whether or not you agree to participate in the study and the study Sponsor accepts no liability for negligence on the part of your hospital’s employees. However, if you are harmed and this is due to someone’s negligence at the hospital, then you may have grounds for a legal action for compensation against the NHS Trust where you are being treated but you may have to pay for your legal costs. The normal National Health Service complaints procedures should be available to you.

Thank you for taking the time to read and consider this information sheet. Should you decide to take part in the study, you will be given a copy of this information sheet and signed consent form to keep.

| *To be completed by the participant:* | |
| --- | --- |
| Once you have read and understood each statement, please enter your initials in each box. | Initial |
| 1. I have read and understood the information sheet for this study. I have had the opportunity to ask questions and have had these answered satisfactorily. |  |
| 1. I understand that participation is voluntary and that I am free to withdraw from the study at any time, without giving a reason, and without my care or legal rights being affected. However, the study team may need to collect some limited information for safety reasons. |  |
| 1. I agree to take part in the above study. |  |
| 1. I give permission for a copy of this fully completed consent form to be sent to the Liverpool Clinical Trials Centre [LCTC] (where it will be kept in a secure location) to allow confirmation that my consent was given. |  |
| 1. I understand that relevant sections of my medical notes and any data collected during the study may be looked at by authorised individuals from the central study team and representatives of the Sponsor, regulatory authorities, and the local NHS Trust. I give permission for these individuals to have access to my records and data. |  |
| 1. I agree to my GP being informed of my participation in the study. |  |
| 1. I agree for the relevant data on my NHS hospital admissions and treatment to be collected from my medical records for the purposes of this study. |  |
| 1. I understand that my data will be kept by the University of Liverpool and at my hospital in a confidential manner for 25 years from the end of the study. |  |
| 1. I consent to samples of my blood to be taken and used for this study. |  |
| 1. I understand the risks associated with X-Rays. |  |
| 1. I consent to having additional X-Ray(s) of my jaw as part of the study. |  |
| 1. I consent to clinical photograph(s) of my jaw to be taken as part of the study. |  |
| The statements below are **optional** (you can still take part in the study even if you do not wish to agree to these): |  |
| **YES NO** | |
| 1. I agree to allow information and data or results arising from this study to be used in future healthcare and/or medical research providing my confidentiality is maintained. |  |
| 1. I consent for samples of my blood to be collected for future research and to be transferred to the University of Liverpool or to other scientists in the UK, or elsewhere. |  |
| 1. I consent for this Consent Form to be transferred along with my blood sample to the University of Liverpool. |  |
| 1. I consent to samples of my blood to be taken and used for future research which may involve genetic testing. |  |

| *To be completed by the participant:* | | | |
| --- | --- | --- | --- |
| Your full name  (please print): |  | | |
| Your signature: |  | Date: |  |
|  | | | |
| *To be completed by the Researcher (after participant has completed the form):* | | | |
| Researcher full name (please print): |  | | |
| Researcher signature: |  | Date: |  |

| Please file the original wet ink copy in the RAPTOR Investigator Site File, and make three copies: one for the participant, one for the medical notes and one to be sent to the LCTC. |
| --- |
