# Supplementary material for: RAPTOR: Randomised Controlled Trial of PENTOCLO (pentoxifylline-tocopherol-clodronate) in Mandibular Osteoradionecrosis—study protocol for an open-label phase II randomised controlled superiority trial
Source: Trials. 2025 Jul 24;26:254. doi: 10.1186/s13063-025-08966-9 (PMC12291413; doi:10.1186/s13063-025-08966-9)
Supplement: Supplementary file 3 — Supplementary Material 3: Appendix 3. RAPTOR GP Letter. [file 13063_2025_8966_MOESM3_ESM.docx]

*<To be printed on trust headed paper>*

**GP Letter**

GP Name

GP Address 1

GP Address 2

GP Address 3

GP Post Code

Dear Dr <<insert GP name >>,

**Re: Patient inclusion in clinical trial - RAPTOR: Randomised Controlled Trial of PENTOCLO* in Mandibular Osteoradionecrosis**

***(Pentoxifylline, Tocopherol & Clodronate)**

**Patient name**:	…<<Insert Patient Name >>…………………………..

**Date of Birth**:	…<<Insert Patient DOB>>……………………………..

**NHS Number**:	…<<Insert Patient NHS Number>>…………………

After giving written informed consent, the above patient has been entered into the above named clinical trial. Please find enclosed a copy of the Patient Information Sheet & Consent Form (PISC) for the study containing all the relevant information.

RAPTOR is a multi-centre, unblinded, randomised, phase II trial, with blinded assessment of primary endpoint, with a 1:1 allocation ratio.

RAPTOR aims to establish the value of the repurposed drug combination Pentoxifylline, Tocopherol & Clodronate (termed PENTOCLO) in treating osteoradionecrosis of the mandible.

The target population is patients who have previously been cured of head and neck cancer that have received radiotherapy, and who have osteoradionecrosis of the mandible.

As part of the trial, patients will be randomly allocated to one of two groups:

1) Arm A – Control Arm: Standardised supportive care (SSC), or

2) Arm B – Experimental Arm: SSC and PENTOCLO

Add additional detail for GP here

If you require further details please contact: ______________________________________________

Yours sincerely,
